# A Potent Class of GPR40 Full Agonists Engages the EnteroInsular Axis to Promote Glucose Control in Rodents

**DOI:** 10.1371/journal.pone.0046300

**Published:** 2012-10-09

**Authors:** Jian Luo, Gayathri Swaminath, Sean P. Brown, Jane Zhang, Qi Guo, Michael Chen, Kathy Nguyen, Thanhvien Tran, Lynn Miao, Paul J. Dransfield, Marc Vimolratana, Jonathan B. Houze, Simon Wong, Maria Toteva, Bei Shan, Frank Li, Run Zhuang, Daniel C.-H. Lin

**Affiliations:** 1 Department of Metabolic Disorders, Amgen Inc., South San Francisco, California, United States of America; 2 Department of Therapeutic Discovery, Amgen Inc., South San Francisco, California, United States of America; 3 Department of Pharmacokinetics and Drug Metabolism, Amgen Inc., South San Francisco, California, United States of America; 4 Department of Pharmaceutics, Amgen Inc., South San Francisco, California, United States of America; University of British Columbia, Canada

## Abstract

Type 2 diabetes is characterized by impaired glucose homeostasis due to defects in insulin secretion, insulin resistance and the incretin response. GPR40 (FFAR1 or FFA1) is a G-protein-coupled receptor (GPCR), primarily expressed in insulin-producing pancreatic β-cells and incretin-producing enteroendocrine cells of the small intestine. Several GPR40 agonists, including AMG 837 and TAK-875, have been disclosed, but no GPR40 synthetic agonists have been reported that engage both the insulinogenic and incretinogenic axes. In this report we provide a molecular explanation and describe the discovery of a unique and potent class of GPR40 full agonists that engages the enteroinsular axis to promote dramatic improvement in glucose control in rodents. GPR40 full agonists AM-1638 and AM-6226 stimulate GLP-1 and GIP secretion from intestinal enteroendocrine cells and increase GSIS from pancreatic islets, leading to enhanced glucose control in the high fat fed, streptozotocin treated and NONcNZO10/LtJ mouse models of type 2 diabetes. The improvement in hyperglycemia by AM-1638 was reduced in the presence of the GLP-1 receptor antagonist Ex(9–39)NH_2_.

## Introduction

GPR40 is a 7-transmembrane receptor that responds to a plethora of long-chain fatty acids [1], [2]. GPR40 is expressed on gut enteroendocrine cells [3], [4] and pancreatic β-cells [1], [5], key cell types that respond to nutrients and modulate whole body glucose homeostasis. Stimulation of the GPR40 pathway in these cells results in elevated serum levels of the incretins GLP-1 (glucagon-like peptide 1) and GIP (glucose-dependent insulinotropic polypeptide), and insulin. Mimetics and secretagogues of insulin are a mainstay of the diabetic pharmacopeia while mimetics and stabilizers of GLP-1 are gaining increased acceptance [6], [7]. By combining the activity along both of these axes into one molecular entity, synthetic agonists that target GPR40 represent a novel approach for glycemic control. However, to date, no GPR40 agonists have been described which both increase incretin levels and insulin levels. We describe the discovery and characterization of a novel class of GPR40 full agonists with improved pharmacology compared to the clinical candidate, AMG 837.

GPR40 has gained considerable interest as a target for type 2 diabetes from pharmaceutical companies, investigators and the medical community [8], [9]. Because the actions of GPR40 to stimulate insulin secretion from pancreatic β-cells are dependent on elevated glucose levels [1], the risk of hypoglycemic episodes following GPR40 agonist treatment is low. In rodents, deletion of GPR40 results in reduced insulin levels in response to both acute lipid stimuli [10] and after long-term high fat feeding [11]. Additionally, GPR40 null mice displayed a reduced incretin response to free fatty acids [3] and impaired glucose and arginine induced insulin secretion *in vivo* [12]. In humans, a GPR40 variant (Gly180Ser) was described which was linked to a reduced insulin secretory capacity [13]. These studies support efforts to discover novel GPR40 agonists for therapeutic use in type 2 diabetes.

Previous disclosures have described the discovery of orally bioavailable GPR40 agonists that displayed efficacy in a variety of rodent diabetic models without exhibiting hypoglycemia [14]–[17]. Two of these molecules, AMG 837 [17], [18] and TAK-875 [16], [19], were selected for further clinical evaluation as antihyperglycemic agents. TAK-875 was found to improve glycemic parameters in type 2 diabetic subjects, but not in healthy volunteers [19]. Similarly, in phase 1 clinical trials in healthy volunteers, AMG 837 did not lower glucose or increase insulin levels (Amgen, data on file). While both agonists enhance insulin secretion from pancreatic β-cells and lower post-prandial glucose in non-clinical models, neither these nor other reported synthetic GPR40 agonists have been shown to significantly improve incretin levels *in vivo*, including in phase 2 clinical trials of 12-weeks in duration with TAK-875 [20], [21]. GPR40 full agonists, discovered and described here, represent a unique class of GPR40 agonists with greater glucose lowering efficacy in rodents.

## Results

### Discovery of AM-1638 and AM-6226

**Figure 1:**
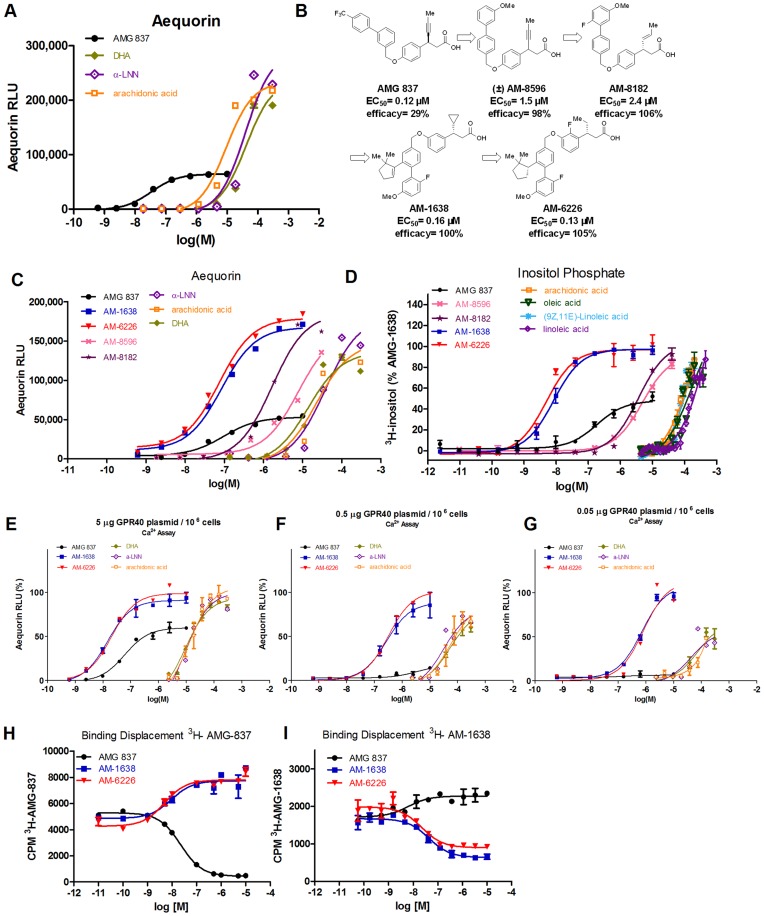
*In vitro* characterization of AM-1638 and AM-6226 and comparison to AMG 837. (A) Aequorin Ca^2+^ assay comparing AMG 837 to natural fatty acid ligands DHA, α-LNN and arachidonic acid. (B) Chemical structures of the key compounds synthesized during the medicinal chemistry effort that led to the discovery of AM-1638 and AM-6226. (C) Aequorin Ca^2+^ flux with key synthetic agonists and fatty acids. (D) Inositol phosphate assay with key synthetic agonists and fatty acids. (E–G) Plasmid titration experiments to examine agonist activity under conditions with reduced receptor levels, where either 5000 ng (E), 500 ng (F) or 50 ng (G) of GPR40 (FFAR1) expression plasmid was co-transfected with aequorin expression plasmids into CHO cells. (H) Competition binding experiment with ^3^H-AMG 837. (I) Competition binding experiment with ^3^H-AM-1638.

Characterization of AMG 837 in an assay monitoring calcium flux in CHO cells (a CHO cell line stably expressing GPR40) revealed that it displays partial agonist activity against GPR40 (figure 1A, AMG 837 EC_50_ = 0.12±0.01 µM, n = 26, 29% E_max_) compared to the natural free fatty acid ligands, confirming previously published results of partial agonism [17]. In another GPR40 stable cell line derived from A9 cells (A9_GPR40) [17], the maximal activity of AMG-837 was 2-fold lower than that of several fatty acid natural ligands, including arachidonic, oleic, and linoleic acids (figure 1D). We confirmed that other published synthetic agonists, GW-9508 [22] and TUG-424 [23] were also partial agonists (figure S1). Since GPR40 agonists with greater intrinsic activity may have improved anti-diabetic properties, we focused our efforts to discover potent synthetic full agonists.

Further examination of our collection of GPR40 agonists synthesized during the discovery of the partial agonist AMG 837 [18] with this CHO-GPR40 stable cell line provided us with our initial full agonist lead (±)-AM-8596 (figure 1B,C; EC_50_ = 1.5±0.17 µM, n = 15, 98% E_max_). Interestingly, evaluation of the single enantiomers of this full agonist lead (±)-AM-8596 revealed that the (*S*)-AM-8596 enantiomer was a partial agonist (EC_50_ = 0.65±0.03 µM, n = 5, 30% E_max_) while the less potent enantiomer, (*R*)- AM-8596, displayed the desired properties of full agonism (EC_50_ = 3.8±0.54 µM, n = 10, 98% E_max_), prompting us to explore this chemical lead for further optimization of its properties.

**Figure 2:**
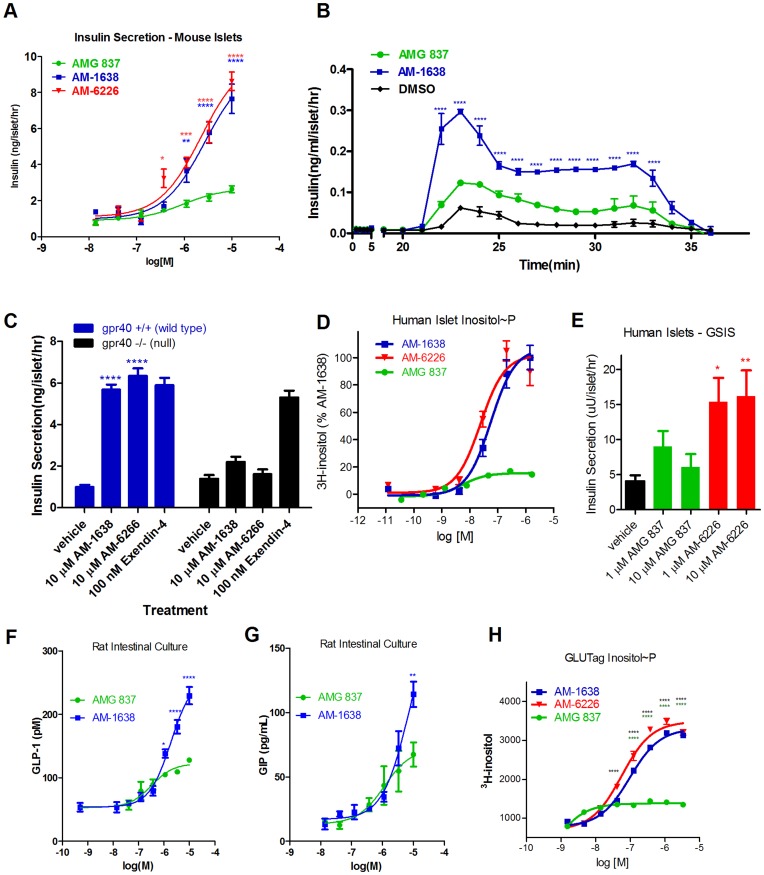
Activity of AM-1638 and AM-6226 in primary cells. (A) GSIS assay in mouse islets incubated with a dose response of compounds in the presence of 16.7 mM glucose. (B) Mouse islet perifusion assay. The glucose concentration was raised from 3 mM to 16.7 mM at t = 20 minutes and returned to 3 mM glucose at t = 30 minutes. 10 µM compound was perifused through the entire experiment. (C) GSIS assay with islets from wild type or GPR40 knock-out mice. (D) Inositol phosphate assay with dispersed human islet cells. (E) GSIS assay with human islets incubated with GPR40 agonists and 12.5 mM glucose. (F) GLP-1 secretion assay with fetal rat intestinal cells. (G) GIP-1 secretion assay with fetal rat intestinal cells. (H) Inositol phosphate accumulation assay using the mouse GLUTag enteroendocrine L-cell line. Statistical significance is denoted by *(p<0.05), **(p<0.01), ***(p<0.001) and ****(p<0.0001), as determined by one-way or two-way ANOVA, and are color-coded to treatment in the figure legends. For (A), (B), (F), (G), and (H) statistical comparisons were made to AMG 837 treatment. For (C) and (E) statistical comparisons were made to vehicle treatment.

Addition of a fluoro substituent to the terminal aryl ring and exchange of the propynyl moiety of (*R*)-AM-8596 for a *trans*-1-propenyl group provided AM-8182 that displays a modest increase in potency and maintains full agonism (Figure 1B,C; EC_50_ = 2.4±0.1 µM, n = 16, 106% E_max_). Three further chemical modifications: exchange of the *trans*-propenyl group for a cyclopropane, relocation of the ether linkage from a *para*- to an *ortho*-orientation on the central phenyl ring, and addition of a 5,5-dimethylcyclopent-1-enyl group to the biphenyl moiety led to the discovery of the potent full agonist AM-1638 (Figure 1B, C; 0.16±0.01 µM, n = 21, 100% E_max_). Further optimization of AM-1638 resulted in the discovery of AM-6226 (Figure 2B, C; EC_50_ = 0.13±0.03 µM, n = 21, 105% E_max_).

### Characterization of AM-1638 and AM-6226 *in vitro*

We characterized AM-1638 and AM-6226 in a number of assays to confirm their properties. The second messenger inositol phosphate was measured in response to the leads as well as AM-1638 and AM-6226; both agonists had intrinisic activity similar to the endogenous fatty acid ligands with ∼2-fold greater intrinsic activity than AMG 837 (figure 1D). Half-maximal potency in this assay format was 13.1±1.1 nM (n = 12) and 5.7±0.8 nM (n = 11) for AM-1638 and AM-6226, respectively. The greater potency of AM-1638 and AM-6226 in the inositol phosphate assay compared to the aequorin Ca^2+^ flux assay is likely due to the fact that that the inositol phosphate assay measures second messenger levels accumulated over an 1-hour period, while the aequorin assay measures Ca^2+^ levels over a 20-second interval immediately after addition of compounds.

Transient transfection of GPR40 at reduced levels allows for clear differentiation of full and partial agonists [17]. CHO cells were transiently transfected with aequorin and 10-fold dilutions of GPR40 expression plasmid in parallel, but the total amount of plasmid DNA was kept constant by adding the appropriate amount of empty vector DNA. In this assay format, AM-1638 and AM-6226 displayed greater intrinisic efficacy than AMG 837 and this difference magnified at reduced receptor expression levels. The maximal efficacy of AMG 837 was ∼60%, ∼20% and ∼5% of the maximal efficacy of AM-1638 and AM-6226 when 5, 0.5 and 0.05 µg of GPR40 expression plasmid were transfected, respectively (figures 1E, F, and G).

Competition binding experiments using radiolabelled AMG 837 or AM-1638 were performed. Unlabelled AMG 837 displaced ^3^H-AMG 837, but not ^3^H-AM-1638. Conversely, unlabelled AM-1638 or AM-6226 displaced ^3^H-AM-1638, but not ^3^H-AMG-837. These results revealed that AM-1638 and AM-6226 binds to a site distinct from that which AMG 837 binds (figure 1 H,I), further underscoring the unique properties of this set of agonists. Additionally, AMG 837 and AM-1638/AM-6226 act as positive allosteric modulators to one another and those data are the subject of an additional report [24].

To explore the specificity of AM-1638 and AM-6226, we tested their activity on receptors related to GPR40. In the aequorin assay, AM-1638 and AM-6226 did not activate the receptors GPR41 (FFAR3) and GPR43 (FFAR2) up to 10 µM, but had weak activity on the lipid activated receptor GPR120 (EC_50_ ∼5 µM) (figure S2). Both agonists did not activate PPAR alpha, delta, or gamma at concentrations up to 10 µM (figure S2).

### Stimulation of Second Messengers and Secretion of Insulin, GLP-1 and GIP in Primary Cells

The divergent pharmacology displayed by GPR40 full agonists AM-1638 and AM-6226 from AMG 837 motivated us to elucidate these compounds’ effects on isolated primary cells, which would more closely mimic the endogenous receptor expression levels. Mouse islets were isolated and potentiation of insulin secretion at 16.7 mM glucose was tested with different doses of AMG 837, AM-1638 and AM-6226. Both AM-1638 and AM-6226 increased insulin secretion 3–4 fold from that of AMG 837 (figure 2A). The potency of AM-1638 and AM-6226 in this assay was EC_50_ = 0.99±0.15 µM (n = 6) and EC_50_ = 0.94±0.6 µM (n = 3), respectively. Because AM-1638 and AM-6226 had overall similar properties in recombinant-cell expressing GPR40 functional assays and mouse islets, we focused most of the subsequent experiments on AM-1638.

To understand the effects on the kinetics of insulin secretion, AM-1638 and AMG 837 were tested at 10 µM in an islet perifusion system in the presence of both 3 mM and 16.7 mM glucose. Under conditions containing 3 mM glucose, neither AMG 837 nor AM-1638 stimulated insulin compared to the vehicle control (figure 2B). In the presence of 16.7 mM glucose, AM-1638 and AMG 837 increased both first and second phase insulin secretion, but the total amount of insulin secreted was greater for AM-1638 in both phases (t = 20–30 minutes, figure 2B). That AM-1638 did not potentiate GSIS at 3 mM glucose indicates that the glucose-dependent aspects of AM-1638 on pancreatic β-cell insulin secretion were maintained. These effects were specific to GPR40, as islets isolated from GPR40 knockout mice did not respond to AM-1638 or AM-6226, but did respond to Exendin-4, which exerts its effects via GLP-1R (figure 2C).

These results were extended to isolated primary human islets cells. Measuring the accumulation of the second messenger inositol phosphate in response to agonist treatment, we observed that both AM-1638 and AM-6226 potently stimulated IP production in human islets with ∼8–10×greater efficacy than AMG 837 (figure 2D). Stimulation of insulin secretion at 12.5 mM glucose was also significantly greater for AM-6226 than AMG 837 (figure 2E), suggesting again the potential therapeutic use of these molecules with greater anti-diabetic properties.

Increased GLP-1 and GIP would be expected to have additional benefits in type 2 diabetics [25]. While GPR40 is co-expressed with both GLP-1 and GIP in gut enteroendocrine cells [3], [4], no synthetic GPR40 agonists have been shown to induce secretion of these peptide hormones. We tested for potential effects on incretin secretion *in vitro* by using cells isolated from fetal rat intestines [26]. Dose responses measuring GLP-1 and GIP secretion into the culture media following incubation with AM-1638 and AMG 837 demonstrated that both agonists could stimulate GLP-1 release *in vitro* but the magnitude of effect was greater with AM-1638 (figure 2F,G). The murine enteroendocrine GLUTag cell line expresses GPR40[27], and in an inositol phosphate accumulation assay with GLUTag cells, AM-1638 and AM-6226 had markedly enhanced activity (3–4 fold increase) compared to AMG 837 (figure 2H). Taken together, these results demonstrate greater insulin and incretin stimulation by AM-1638/AM-6226, indicating that this class of agonists may have enhanced anti-hyperglycemic activity *in vivo* compared to the class represented by AMG 837.

### Enhanced Anti-diabetic Efficacy of AM-1638 in Rodent Models

The impressive *in vitro* profile of AM-1638 warranted further examination in diabetic rodent models *in vivo*. AM-1638 possessed pharmacokinetic properties suitable for oral dosing in the rat, mouse, beagle dog and cynomolgus monkey [28]. We first evaluated *in vivo* pharmacology in the high-fat fed, low-dose streptozotocin-treated mouse model of type 2 diabetes [29]. This model was chosen since it features both insulin resistance and reduced β-cell capacity, two hallmarks of type 2 diabetes.

**Figure 3:**
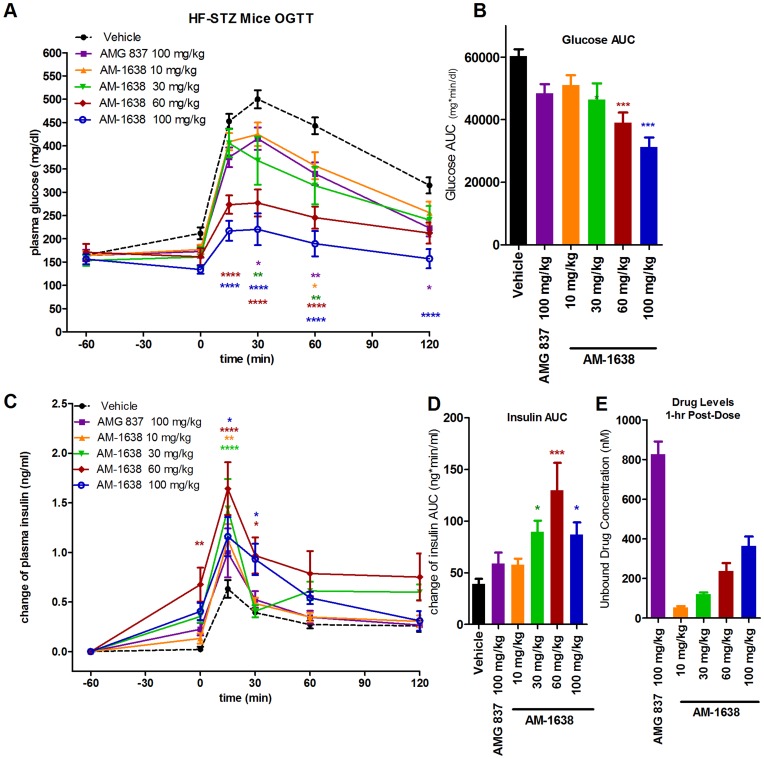
Enhanced *in vivo* efficacy of AM-1638 compared to AMG 837 in HF/STZ type 2 diabetic mice. Drug treatment was administered 1-hour prior to an oral glucose bolus. (A) Glucose levels during an OGTT in high-fat fed, streptozotocin treated type 2 diabetic mice. (B) Glucose AUC values. (C) Change in plasma insulin levels from baseline during an OGTT (D) Insulin AUC (E) Unbound (free) plasma drug concentration in plasma 1-hour following drug dose, as determined by MS. Statistical significance compared to vehicle treatment is denoted by *(p<0.05), **(p<0.01), ***(p<0.001) and ****(p<0.0001), as determined by one-way or two-way ANOVA, and are color-coded to the treatment in the figure legends.

AM-1638 was dosed at 10, 30, 60 and 100 mg/kg followed by an oral glucose tolerance test (OGTT). Glucose AUC following improved 15, 23, 35 and 48% at 10, 30, 60 and 100 mg/kg AM-1638, respectively (figure 3A,B). These effects were compared in parallel to AMG 837; preliminary experiments indicated that AMG 837 was maximally efficacious at doses ≥60 mg/kg in this model. Following a single 100 mg/kg dose, AMG 837 improved post-challenge glucose AUC by 19% (figure 3A,B). Thus, the greater intrinsic activity of AM-1638 *in vitro* translated to improved glucose control *in vivo*.

In order to understand the mechanisms underlying the improved glucose clearance, we measured insulin levels in response to AM-1638 treatment. AM-1638 at the higher doses stimulated greater insulin secretion 1.5–2.2 fold greater than AMG 837 (figure 3C,D). In order to verify that sufficient drug levels of both AM-1638 and AMG-837 were present, we measured plasma drug levels at the time of glucose challenge (1-hour post drug administration). The free drug level in plasma at 100 mg/kg AM-1638 and AMG 837 were 363±48 and 826±66 nM, respectively (figure 3E) which exceeded the EC_50_ values for these agonists on the mouse GPR40 inositol phosphate accumulation assay (AM-1638: EC_50_ = 12.9±1.4 nM (n = 2); AMG 837: 11.0±0.05 nM (n = 2)).

**Figure 4:**
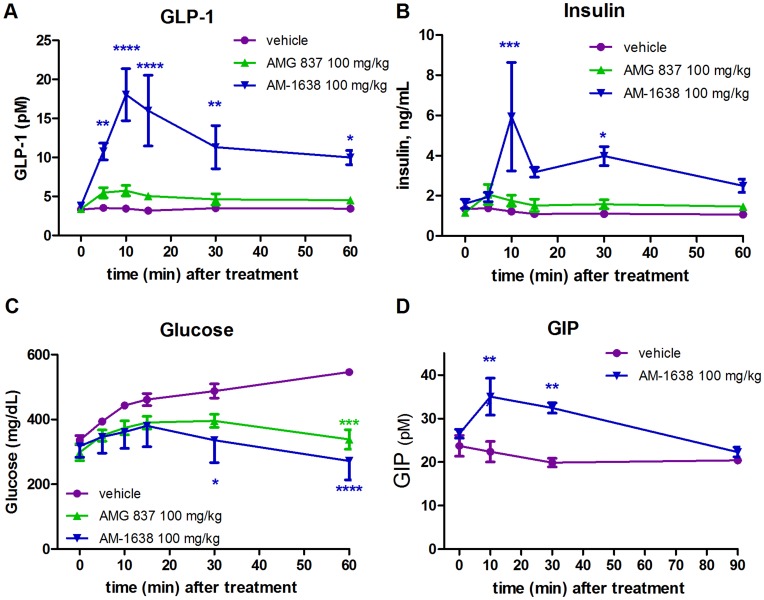
Engagement of the enteroendocrine axis in HF/STZ type 2 diabetic mice by AM-1638. Following administration of a single dose of the indicated treatments at t = 0 minutes in HF/STZ mice, measurements of (A) GLP-1 (B) insulin (C) glucose and (D) GIP were taken at various timepoints. Statistical significance compared to vehicle treatment is denoted by *(p<0.05), **(p<0.01), ***(p<0.001) and ****(p<0.0001), as determined by one-way or two-way ANOVA, and are color-coded to the treatment in the figure legends.

The increase in insulin secretion by GPR40 agonists is due to direct stimulation of GPR40 receptors on pancreatic β-cells, but may also be derived from stimulation of the gut peptides GLP-1 and GIP, which subsequently act on the GLP-1 and GIP G protein-coupled receptors found on pancreatic β-cells. AM-1638 and AMG 837 were also tested for stimulation of secretion of the incretins GLP-1 in HF/STZ mice. A single dose of AM-1638 or AMG 837 was administered by oral gavage, and blood GLP-1, insulin and glucose levels were measured at various time-points following dosing. No glucose challenge was performed in order to isolate the effects of the compounds alone. The full agonist AM-1638 differentiated itself from the AMG 837 by exhibiting a markedly greater increase in both plasma GLP-1 and insulin levels (figure 4A,B). While plasma glucose levels trended higher in vehicle treated animals, stabilized blood glucose levels were observed in both the AMG 837 and AM-1638 treated groups (figure 4C). AM-1638 also stimulated secretion of GIP following a single oral dose (figure 4D). The increase in GLP-1 and GIP by AM-1638 likely contributed to the enhanced insulin response observed during the glucose challenge (figure 3C,D).

### AM-1638 Enhances Incretin and Insulin Secretion in NONcNZO10/LtJ Mice

**Figure 5:**
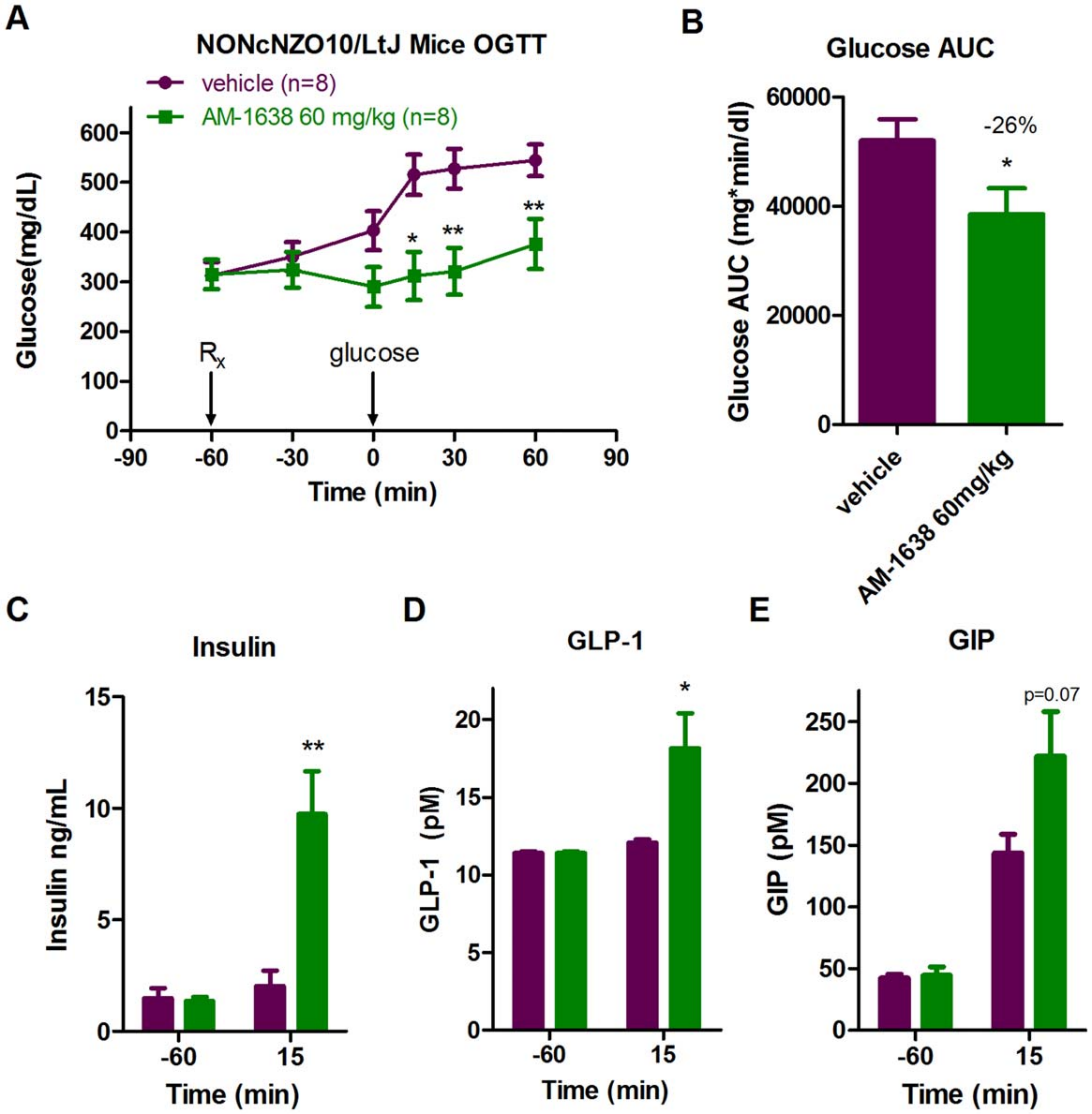
During an OGTT in NONcNZO10/LtJ type 2 diabetic mice, AM-1638 lowers blood glucose levels through an increase in insulin and incretin secretion. Vehicle (purple, n = 8) or 60 mg/kg AM-1638 (green, n = 8) was administered 1-hour prior to an oral glucose bolus. (A) Glucose levels at various timepoints. (B) Glucose AUC values. (C) Plasma insulin levels (D) GLP-1 levels and (E) GIP levels at baseline (-60 minutes) and 15 minutes after glucose challenge. Statistical significance compared to vehicle treatment is denoted by *(p<0.05), **(p<0.01), ***(p<0.001) and ****(p<0.0001), as determined by two-way ANOVA or student’s t-test.

In order to confirm and expand these results to a different rodent type 2 diabetes model, we tested the effects of AM-1638 in the NONcNZO10/LtJ (RCS10) mouse model of diabetes. NONcNZO10/LtJ mice retain an intact leptin pathway but contain polygenic defects contributing to moderate and mature onset obesity and diabetes [30], [31]. Treatment with a single 60 mg/kg oral dose of AM-1638 resulted in almost complete suppression of glucose excursions during an oral glucose tolerance test (figure 5A,B). Glucose AUC was reduced 26% when compared to that with vehicle treatment (figure 5B). Similar to that observed in high-fat fed, low-dose streptozotocin-treated mice, both insulin and GLP-1 levels 15 minutes after glucose challenge were significantly increased in AM-1638 treated NONcNZO10/LtJ mice when compared to vehicle treated animals and baseline levels (figure 5C,D). GIP levels appeared elevated in AM-1638 treated animals, but when compared to vehicle treated mice this value did not reach statistical significance (figure 5E). Taken together, these results demonstrate that AM-1638 acts on both intestinal L-cells and pancreatic β-cells to ultimately stimulate insulin secretion and improve glucose homeostasis.

### Evaluation of AM-1638 in GPR40 Null Mice or in the Presence of GLP-1 Receptor Antagonist Exendin(9–39)NH_2_

**Figure 6:**
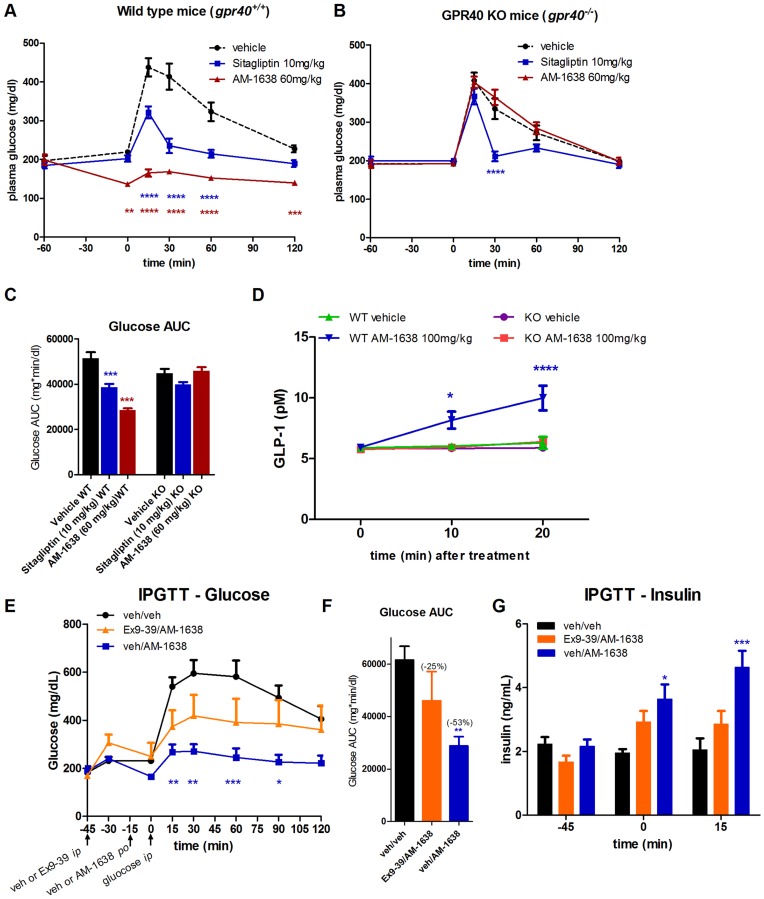
Specificity of AM-1638 to GPR40 (FFAR1) *in vivo* and effect of the GLP-1R antagonist GLP-1(9–39)NH_2_. An OGTT was performed in (A) wild type or (B) GPR40 null mice following a single oral dose of AM-1638 or sitagliptin. Glucose was dosed 1-hr post drug treatment. (C) Glucose AUC during OGTT. (D) GLP-1 secretion following a single oral dose of AM-1638 in wild type or GPR40 null mice. AM-1638 (60 mg/kg) was tested in an IPGTT in the presence or absence of the GLP-1R antagonist GLP-1(9–39)NH_2_ (300 µg/kg) as described in the Materials and Methods section. (E) Plasma glucose levels (F) Glucose AUC and (G) plasma insulin levels at the indicated timepoints during the experiment. Statistical significance compared to vehicle treatment is denoted by *(p<0.05), **(p<0.01), ***(p<0.001) and ****(p<0.0001), as determined by one-way or two-way ANOVA, and are color-coded to the treatment in the figure legends.

GPR40 null mice were used to understand whether the effect of AM-1638 was mediated through GPR40 *in vivo*. Wild type and GPR40 knockout mice were tested in an OGTT following a single dose of AM-1638 or sitagliptin, which exerts its effects via inhibition of DPP-IV [32]. The improvement in pre- (before glucose-bolus) and post-challenge glucose levels were evident in wild type but not GPR40 knockout mice, while the effect of sitagliptin was maintained in both (figure 6A,B,C). Agonism of GPR120 has been reported to mediate GLP-1 secretion [33], [34], and because AM-1638 and AM-6226 have weak activity on GPR120 (EC_50_ ∼ 5 µM, figure S2), it was important to determine whether the increase in GLP-1 *in vivo* was dependent on GPR40. A single dose of AM-1638 at 100 mg/kg elicited an increase in plasma GLP-1 levels in wild type but not GPR40 knockout mice (figure 6D). *In toto*, these results indicate that the pharmacology of AM-1638 is specific to GPR40 *in vivo*.

In order to understand the contribution of the GLP-1 pathway to the improvement in glycemia observed with AM-1638, we tested the effect of the GLP-1R antagonist Exendin(9–39)NH_2_ [35], [36] on AM-1638 activity. Exendin(9–39)NH_2_ was dosed 15 minutes prior to dosing with either vehicle or AM-1638 in wild type C57/Bl6 mice. An intraperitoneal rather than oral glucose challenge was performed in order to bypass the incretin effect of an oral glucose administration. AM-1638 alone resulted in a marked improvement in glucose levels and an increase in insulin levels, but both of these effects were blunted in the presence of Exendin(9–39)NH_2_ (figure 6 E, F, G). Glucose AUC was reduced ∼53% in the presence of AM-1638, but resulted in a ∼25% glucose AUC reduction in the presence of exendin(9–39)NH_2_ (figure 6F)_._ These data indicate that an intact GLP-1R pathway contributes to the efficacy of AM-1638 in this setting.

## Discussion

**Figure 7:**
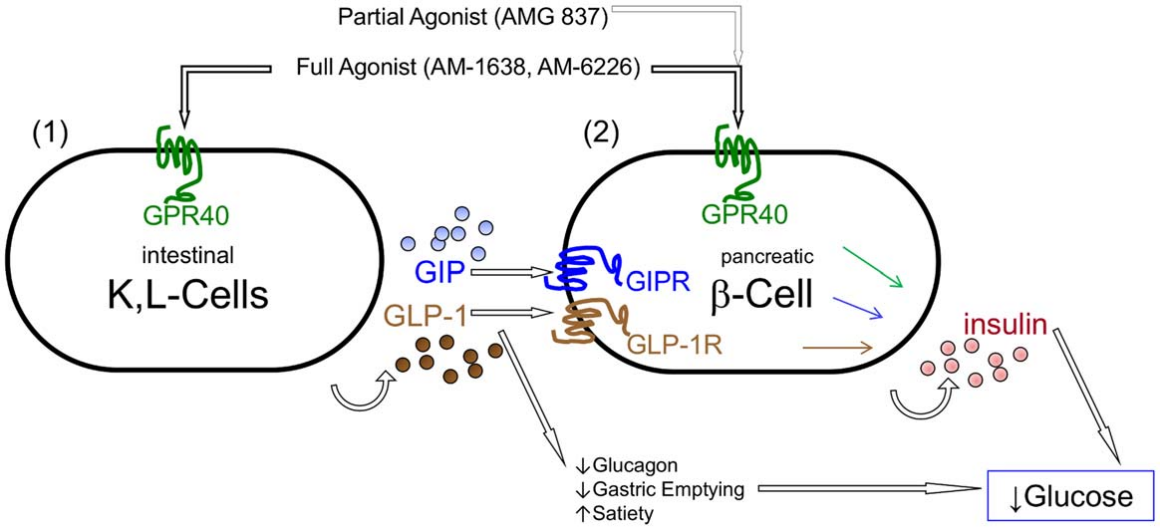
Model depicting the dual mechanism-of-action of GPR40 (FFAR1) full agonists to lower blood glucose levels. GPR40 full agonists engage both the enteroendocrine axis as well as the pancreatic β-cell axis. These pathways both lead to augmentation of glucose stimulated insulin secretion on the pancreatic β-cell. Additionally, GLP-1 has multiple physiological and pharmacological roles, such as inhibition of glucagon secretion, that could further benefit type 2 diabetics. GPR40 partial agonists such as AMG 837 engage only the pancreatic pancreatic β-cell axis *in vivo*.

A new class of GPR40 full agonists, represented by AM-1638 and AM-6226, provides access to a powerful dual mechanism for maintaining glycemic control (figure 7). Two hallmarks of type 2 diabetes are decreased insulin secretion and a reduced incretin effect. GPR40 full agonists present an opportunity to overcome both of these defects with a single pharmacological agent by increasing insulin secretion in pancreatic β-cells, and inducing GLP-1 and GIP secretion from intestinal enteroendocrine cells. GPR40 partial agonists, such as AMG-837, appear to engage the single pancreatic β-cell axis to increase GSIS and lower prandial glucose levels (figure 7). AM-1638 is the first synthetic GPR40 agonist that is reported to increase incretin levels in non-clinical models *in vivo*. Thus, GPR40 full agonists could benefit from the additional actions of GLP-1, which include suppression of glucagon levels, increased satiety, weight loss and beneficial effects on the cardiovascular system [25], [37].

In contrast to AM-1638, the partial agonist AMG 837 does not substantially increase GLP-1 or GIP levels *in vivo*. This is not likely due to a lack of exposure of AMG 837 to the enteroendocrine cells in the gut, as the compound was dosed by oral gavage at doses as high as 100 mg/kg. Possibly, there are lower GPR40 receptor levels on enteroendocrine cells compared to islet β-cells and/or the cellular mechanisms that promote incretin secretion are unique from those that promote insulin secretion. In this context, only a GPR40 full agonist, such as AM-1638 or the fatty acid natural ligands, can appropriately engage GLP-1 and GIP secretion. The GLP-1 pathway contributes to the enhanced efficacy of AM-1638 since the improvement in glycemia was reduced in the presence of the GLP-1R antagonist (figure 6E–G). Overall, the greater *in vivo* efficacy of AM-1638 compared to AMG 837 is likely a result of enhanced stimulation of both the enteroendocrine and β-cell axes. Consistent with this, in isolated settings, such as in the mouse L-cell line GLUTag or with purified human or rodent islets, AM-1638 and AM-6226 stimulated a greater second messenger response compared to AMG 837 (figure 2).

Combination therapy is thought to provide improved glycemic control due to the ability of multiple therapies to influence several diabetic defects. Full agonists of GPR40 offer a unique dual-mechanism to address deficiencies in both insulin and incretin release. Based on the mechanism-of-action, combination of GPR40 full agonists with a DPP-IV inhibitor may provide even greater glucose control by stabilizing elevated GLP-1 levels. Further, we expect GPR40 full agonists to potentially also induce secretion of other gut hormones, such as peptide YY and cholecystokinin [38], [39], which may exert additional benefits for type 2 diabetic patients.

We expect that other partial agonists with intrinsic activity equivalent to that of AMG 837, such as GW-9508[22] and TUG-424[23], will have similar pharmacological properties to AMG 837, when properly controlled for drug exposure. Side-by-side pharmacological experiments of AMG 837 and TAK-875 have not been described, although their chemical structures appear similar (figure S1). Tsujihata *et al* compared TAK-875 to oleic and palmitic acids in a CHO cell line expressing GPR40[40]. While the maximal efficacy appeared similar in that assay, it is possible that the specific CHO cell clone studied overexpresses GPR40, so a difference between partial and full agonists may be masked. Further studies with TAK-875 in systems where GPR40 levels are reduced, such as those described here (*eg* figures 1E, F, G), will be useful. In phase 1 clinical trials in healthy volunteers, both TAK-875[19] and AMG 837 (data on file, Amgen) did not improve glucose levels or increase insulin levels in healthy volunteers. While TAK-875 did improve glycemic parameters in type 2 diabetic patients, these improvements were associated with an increase in plasma insulin but not associated with a change in incretin levels [20], [21]. Based on the enhanced activity and engagement of the incretin axis by the class of molecules defined by AM-1638, greater clinical efficacy may be observed with this class of agonists.

While a single dose of AM-1638 provides clear advantages for glycemic control, important questions remain to be answered. The effects of longer term dosing and sustainability of glycemic improvement by AM-1638/AM-6226 are important areas of investigation. Given that type 2 diabetics often have reduced numbers of functioning pancreatic β-cells, the effects of AM-1638 and AM-6226 or other full agonists on β-cells following long-term dosing is of considerable interest. Partial agonists such as AMG 837 have sustained anti-diabetic effects following 21-day, *qd* dosing in Zucker fatty rats [17] and similar experiments will need to be performed for GPR40 full agonists. Isolated pancreatic islets from both wild type and GPR40 null mice were susceptible to lipotoxicity *in vitro*, suggesting GPR40 is not involved in the ill-effects of prolonged exposures to elevated fatty acids [10]. Nonetheless, prolonged GPR40 agonism may result in both beneficial and harmful consequences on pancreatic β-cells [41]–[43] and will be important to study in an *in vivo* setting. The level of agonism, in terms of both maximal level and duration of exposure, may tip the balance of beneficial and harmful effects in either direction.

While here we have reported the engineering and characteristics of potent, full GPR40 agonists, this research opens doors for targeting GPR40 with other classes of ligands. In recent years the plasticity of GPCR ligand-receptor interactions has gained appreciation [44]. In this light, engineering a GPR40 super-agonist (greater E_max_ than fatty acid ligands) can be envisioned which may result in enhanced glucose control. While tachyphylaxis, loss of glucose-dependence in insulin secretion from islets, or potential harmful consequences on the pancreatic β-cell would continue to be a concern, some of these might be less evident on intestinal enteroendocrine cells given their rapid turnover.

In addition to the enhanced functional activity, AM-1638 and AM-6226 also bind to a distinct site on GPR40 from AMG 837, as determined by competition binding experiments (figure 1H, I). In fact, the AMG 837 and AM-1638 classes of agonists act as positive allosteric modulators to each other as well as the fatty acid natural ligands, and these data are the subject of an additional report [24]. This feature further underscores the uniqueness of this class of molecules and can be further exploited to understand the pharmacology of GPR40. We speculate that combination of the two molecules, either through co-administration or engineered in the form of single molecular entity (dualsteric agonist), may provide additional therapeutic benefit. Additionally, because AM-1638/6226 and AMG 837 can positively cooperate with the fatty acid orthosteric agonists, the activity of AM-1638/6226 and AMG 837 on GPR40 *in vivo* may be influenced by the presence of free fatty acids, and vice versa. Future studies should examine the effect of co-administering AMG 837 and AM-1638/6226 with a lipid infusion or a mixed meal. Additionally, the pharmacology of AMG 837 and AM-1638/6226 on islet insulin secretion *in vivo* as a function of varying serum free fatty acid concentrations and/or different types of free fatty acids is an area of focus.

In conclusion, we have discovered a potent, efficacious class of GPR40 agonists with enhanced efficacy compared to AMG 837. Undoubtedly, these agonists will open new doors of investigation into GPR40 pharmacology and potential therapies for type 2 diabetes.

## Materials and Methods

### GPR40 Agonist Synthesis

AMG 837 and all AM- compounds were synthesized by the Amgen Therapeutics Discovery Department, South San Francisco, CA. Detailed synthesis and analytical properties are included in the Materials and Methods S1 available online. Synthesis of AMG 837 was conducted as described [18]. TUG-424 and GW-9508 were purchased from Tocris Bioscience and Caymen Chemicals, respectively.

### *In vitro* Assays

GPR40 aequorin, plasmid titration, and inositol phosphate assays were performed as described [17]. For aequorin assays, in addition to transient transfection of GPR40 into CHO cells, a CHO cell line stably expressing both GPR40 and aequorin was generated. GLUTag cells were obtained under a license from Dr. Daniel Drucker, University of Toronto. Competition binding experiments were performed using membranes prepared from A9_GPR40 cells [17]. Radio-labeled probes, ^3^H-AMG 837 (specific activity of 52.3 Ci/mmol) and ^3^H-AM-1638 (31.4 Ci/mmol), were custom synthesized at Movarek. Competition binding assays were carried out in 20 mM Hepes pH 7.5, 5 mM MgCl_2_, 100 mM NaCl and 0.1% (w/v) free fatty acid BSA and bound radioligand was collected on 96-well GF/C filter plates.

### Primary Cell Assays

Mouse islets were isolated and tested for stimulation of GSIS as described [17]. Human islets were purchased from Prodo labs or were obtained from the National Institutes of Health and Juvenile Diabetes Research Foundation supported Islet Cell Resource Consortium (http://icr.coh.org). Islet inositol phosphate assays were performed on islets cells that had been dispersed with trypsin. 10,000 islet cells were plated/well of a 96-well plate and were loaded with ^3^H-myo-inositol (0.5 µCi/well) in RPMI overnight prior to treatment. The next day, response to different stimuli was tested in HBSS containing 10 mM LiCl and 0.1% HSA (w/v) for 1 hour at 37°C. Accumulated ^3^H-inositol phosphate was measured after capture to SPA beads. Islet perifusion experiments were performed on mouse islets loaded into a Brandel Suprafusion system. Twenty islets were size-matched and loaded per perifusion chamber. Islets were perifused in KRBH containing, sequentially, 3 mM glucose, then 3 mM glucose containing 10 µM drug, 16.7 mM glucose containing 10 µM drug and finally returned to 3 mM glucose containing 10 µM drug. Insulin was measured using an insulin ELISA kit (Alpco). Fetal rat intestinal cells were isolated from e19-day old fetuses of pregnant Sprague-Dawley rats. Briefly, fetal intestines were isolated and digested in HBSS buffer containing 0.5 mg/ml collagenase (Sigma), 0.5 mg/ml of hyaluronidase (Sigma) and 0.05 mg/ml of deoxyribonuclease I (Roche) for 30 minutes at 37°C. The digestion was quenched using HBSS containing 15% FBS. Fetal rat intestinal cells were washed and cultured overnight in DMEM containing 5% FBS in 96-well plates. To test for secretion of GLP-1 and GIP in response to treatments, we incubated cells for 1 hour with treatments in KRBH buffer containing 0.05% BSA (w/v). GLP-1 and GIP1 secreted into the media was measured using ELISA kits specific for GLP-1 (Linco) or GIP (Millipore).

### Rodent Experiments

All procedures on animals were approved by the Amgen San Francisco Institutional Animal Care and Use Committee (approved protocols #11–04 and #2007–00106). The Amgen San Francisco vivarium is an AAALAC accredited facility. Animals were housed under a 12-h light, 12-h dark cycle (lights-on 0600 h and lights-off 1800 h) and were allowed *ad libidum* access to chow and water. The HF/STZ mice were established as previously described [29]. Briefly, C57BL/6j mice (Jackson labs) at 4 weeks of age were fed a high-fat diet (BioServ S1850, 35% fat) for 3-weeks and then injected with 95 mg/kg streptozotocin (Sigma) *ip* to induce beta cell dysfunction. The animals continued on the same high fat diet for 3–6 additional weeks. Animals were then randomized into treatment groups based on fasting blood glucose and insulin measures. NONcNZO10/LtJ (RCS10) male mice (Jackson labs) were used at 12-weeks of age and randomized for treatment based on fasting glucose levels. For OGTT experiments, drugs were formulated in 1% HPMC, 10% HPbCD, 1% Tween-80 and dosed via oral gavage. Glucose was administered at 2 g/kg 1-hour post drug dose by oral gavage.

IPGTT experiments with Ex(9–39)NH_2_ (Bachem) were performed with male C57BL/6j (n = 6–7/group) at 26-weeks of age. Ex(9–39)NH_2_ was formulated in PBS containing 0.1%BSA and dosed *ip* at 300 µg/kg. Fifteen minutes later, animals were dosed with vehicle or AM-1638 by oral gavage. Glucose was administered by *ip* injection at 2 g/kg 30-minutes following the AM-1638 or vehicle dose.

For all glucose tolerance tests, glucose and insulin measurements were taken from tail vein samples at various time-points. Blood glucose values were measured on an Accu-Chek glucometer (Roche Diagnostics), while insulin levels were determined using an ELISA (Alpco).

### Statistical Analysis

Data were plotted as the mean ± SEM. One-way or two-way ANOVA were carried out using Prism (GraphPad Software) and statistical significance was considered meaningful at p<0.05.

## Supporting Information

Figure S1 **AMG 837, GW-9508 and TUG-424 are partial agonists.** (A) Structure of selected GPR40 agonists. (B) GPR40 agonists were tested in parallel *in vitro* in an inositol phosphate accumulation assay in A9 cells stably expressing GPR40.
(TIF)

Figure S2 **AM-1638 and AM-6226 tested on GPR41, GPR43, GPR120 and PPAR-gamma assays.** AM-1638 and AM-6226 were tested against various receptors in cell-based assays. (A) GPR41 (FFAR3) aequorin assay. GPR41 was force coupled to the calcium signaling pathway by co-transfecting the cells with G_qmyri5_. (B) GPR43 (FFAR2) aequorin assay. The short chain fatty acid propionate acts as a natural agonist ligand for GPR41 and GPR43. (C) GPR120 aequorin assay. The unsaturated fatty acids α-LNN and DHA act as natural agonist ligands for GPR120. (D) PPAR-gamma luciferase assay using the thiazolidinedione rosiglitazone as a positive control. AM-1638 and AM-6226 also did not activate PPAR-alpha and PPAR-delta in luciferase reporter assays in concentrations up to 10 µM (F. Li and DCH Lin, data not shown).
(TIF)

Materials and Methods S1 **Chemical Synthesis of AMG 837, AM-1638, AM-6226, AM-8596, AM-8182, ^3^H-AM-1638 and ^3^H-AMG 837.** Additional materials and methods describing the chemical syntheses of key GPR40 (FFAR1) agonists studied in this report.
(DOC)
