# Supplementary figures and images for: A Potent Class of GPR40 Full Agonists Engages the EnteroInsular Axis to Promote Glucose Control in Rodents
Source: PLoS One. 2012 Oct 9;7(10):e46300. doi: 10.1371/journal.pone.0046300 (PMC3467217; doi:10.1371/journal.pone.0046300)

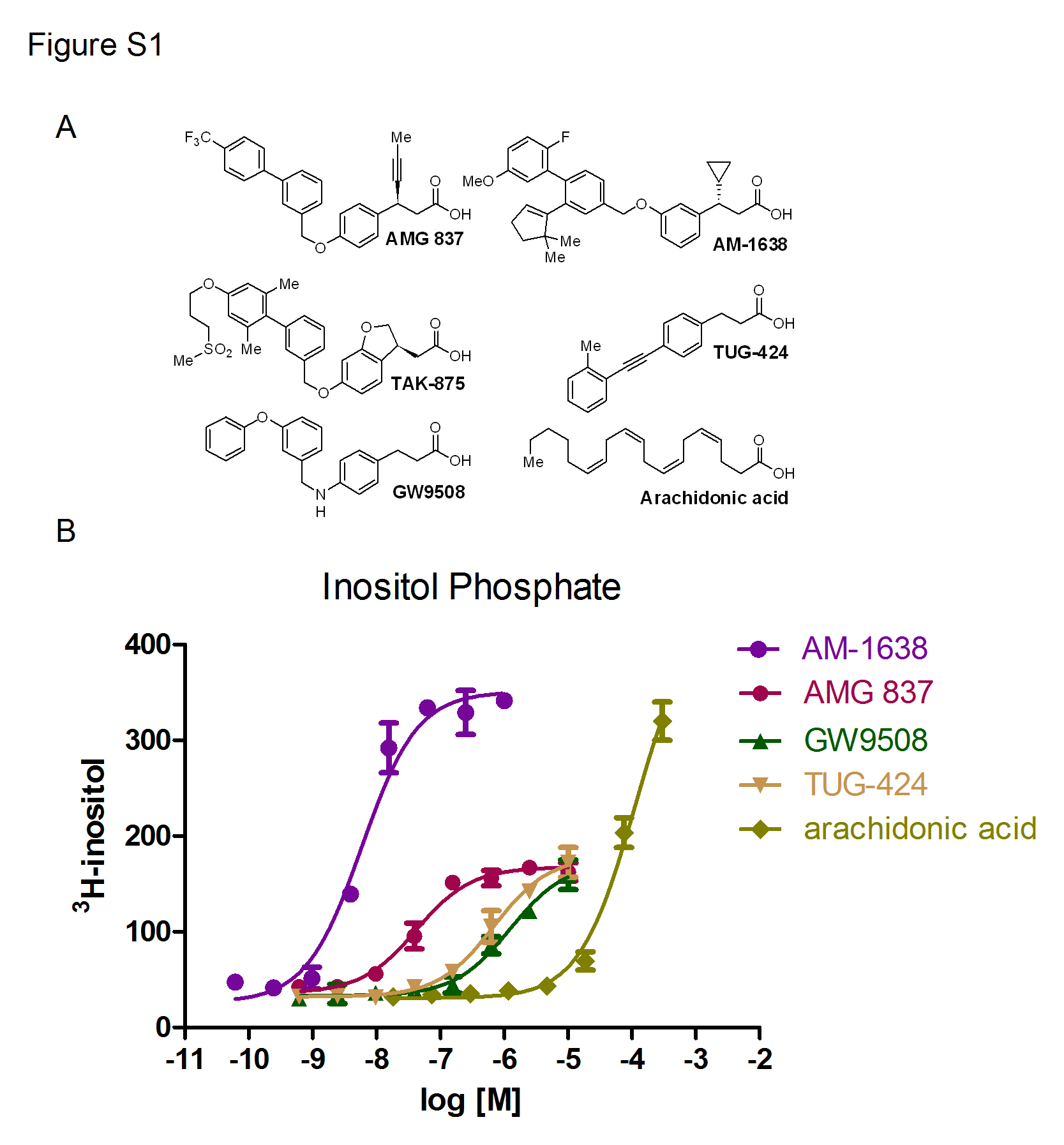

Supplement: Figure S1 — AMG 837, GW-9508 and TUG-424 are partial agonists. (A) Structure of selected GPR40 agonists. (B) GPR40 agonists were tested in parallel in vitro in an inositol phosphate accumulation assay in A9 cells stably expressing GPR40. (TIF) [file pone.0046300.s001.tif]

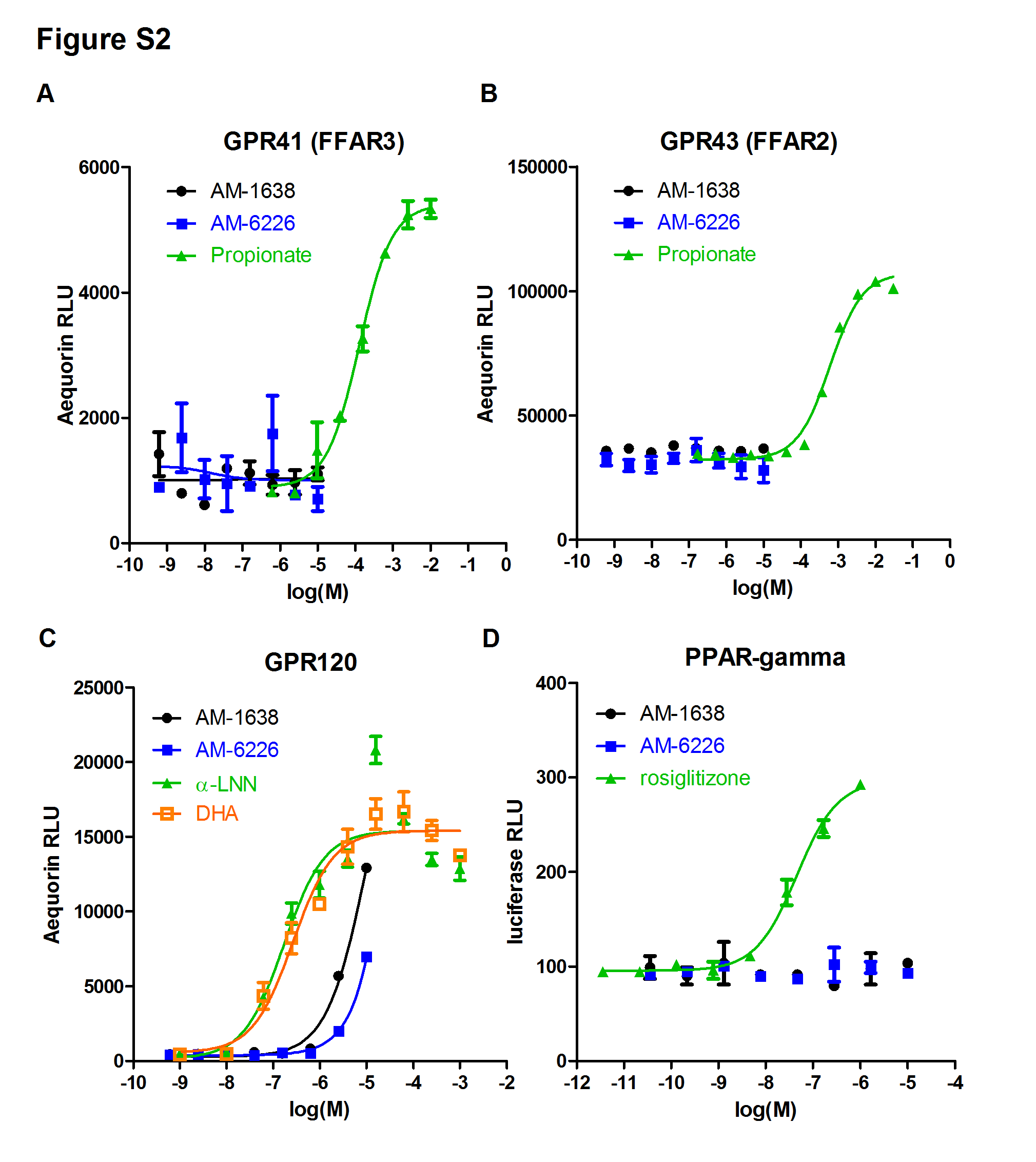

Supplement: Figure S2 — AM-1638 and AM-6226 tested on GPR41, GPR43, GPR120 and PPAR-gamma assays. AM-1638 and AM-6226 were tested against various receptors in cell-based assays. (A) GPR41 (FFAR3) aequorin assay. GPR41 was force coupled to the calcium signaling pathway by co-transfecting the cells with Gqmyri5. (B) GPR43 (FFAR2) aequorin assay. The short chain fatty acid propionate acts as a natural agonist ligand for GPR41 and GPR43. (C) GPR120 aequorin assay. The unsaturated fatty acids α-LNN and DHA act as natural agonist ligands for GPR120. (D) PPAR-gamma luciferase assay using the thiazolidinedione rosiglitazone as a positive control. AM-1638 and AM-6226 also did not activate PPAR-alpha and PPAR-delta in luciferase reporter assays in concentrations up to 10 µM (F. Li and DCH Lin, data not shown). (TIF) [file pone.0046300.s002.tif]
