# Supplementary material for: A Potent Class of GPR40 Full Agonists Engages the EnteroInsular Axis to Promote Glucose Control in Rodents
Source: PLoS One. 2012 Oct 9;7(10):e46300. doi: 10.1371/journal.pone.0046300 (PMC3467217; doi:10.1371/journal.pone.0046300)
Supplement: Materials and Methods S1 — Chemical Synthesis of AMG 837, AM-1638, AM-6226, AM-8596, AM-8182, 3H-AM-1638 and 3H-AMG 837. Additional materials and methods describing the chemical syntheses of key GPR40 (FFAR1) agonists studied in this report. (DOC) [file pone.0046300.s003.doc]

Supplementary Materials and Methods

A Potent Class of GPR40 Full Agonists Engages the EnteroInsular Axis to Promote Glucose Control in Rodents

Jian Luo^a^, Gayathri Swaminath^a^, Sean P. Brown^b^, Jane Zhang^a^, Qi Guo^a^, Michael Chen^a^, Kathy Nguyen^a^, Thanhvien Tran^a^, Lynn Miao^a^, Paul J. Dransfield^b^, Marc Vimolratana^b^, Jonathan B. Houze^b^, Simon Wong^c^, Maria Toteva^d^, Bei Shan^a^, Frank Li^a^, Run Zhuang^a^ and Daniel C.-H. Lin^a,1^

Departments of ^a^Metabolic Disorders, ^b^Therapeutic Discovery, ^c^Pharmacokinetic and Drug Metabolism and ^d^Pharmaceutics, Amgen Inc., 1120 Veterans Boulevard, South San Francisco, CA 94080, United States

^1^ Corresponding author:

Daniel C.-H. Lin

Amgen

Department of Metabolic Disorders

1120 Veterans Blvd.

South San Francisco, CA 94080

[dclin@amgen.com](mailto:dclin@amgen.com)

**Supplementary Materials and Methods – Chemical Synthesis**

**1**

**5-(4-Hydroxy-benzylidene)-2,2-dimethyl-[1,3]dioxane-4,6-dione (1).** A 12L RB flask was charged with 4-hydroxy-benzaldehyde (500 g, 4.09 mol) and water (4.5 L). The flask was heated to 75 °C with a heating mantle and Meldrum’s acid (620 g, 4.30 mmol) was added as a slurry in 2.5 L of water. The reaction mixture was mechanically stirred for 5h at 75 °C then allowed to cool to rt. The product was collected by filtration and rinsed with water. The wet solid was then dissolved in 3.5 L of acetone with heat in a 12L RB flask. The solution was heated to 55°C and 3.5 L of water (at 65°C) was added. Crystallization occurred immediately and stirring was continued until the mixture cooled to rt over 6 hr. The solid was collected by filtration and rinsed with water. After drying thoroughly, 855 g (3.44 mol, 84%) of adduct **1** was obtained as a fine, crystalline yellow powder. ^1^H NMR(500MHz, DMSO-*d*_6_) δ ppm 9.75 (br s, 1H), 8.27 (s, 1H), 8.24 (d, 2H, *J*=10 Hz), 6.98 (d, 2H, *J*=10 Hz), 1.76 (s, 6H). MS ESI (pos.) m/e: 519.0 (2M + Na).

**1 2**

**(+/-)-5-[1-(4-Hydroxy-phenyl)-but-2-ynyl]-2,2-dimethyl-[1,3]dioxane-4,6-dione (2).** A 12L 3-neck flask was equipped with a mechanical stirrer, nitrogen inlet, nitrogen outlet and placed in a room-temperature water bath. After purging with nitrogen for 2 hr, a solution of 1-propynylmagnesium bromide in THF (0.5 N, 5 L) was added by cannula. In a separate nitrogen-flushed 5 L RB flask, compound **6.1** (200 g, 805 mmol) was dissolved in anhydrous THF (2.2 L) with gentle warming. The solution of **6.1** was then added over 15 minutes. Over the course of the addition, the reaction mixture changed to a thick, yellow suspension. After the addition was complete, the reaction mixture was stirred for 15 minutes and then quenched with aqueous NH_4_Cl (1 M, 2 L) and diluted with hexanes (1.5 L). The layers were separated and the organic layer discarded. The aqueous layer was acidified to pH ~2 with Conc. HCl and extracted with 2-pentanone (2 L). The extract was washed with water, and concentrated to a volume of 1L for the next reaction. ^1^H NMR(500MHz, acetone-*d*_6_) δ ppm  8.26 (s, 1H), 7.39 (d, 2H, *J*=8.5 Hz), 6.76 (d, 2H, *J*=8.4 Hz), 4.73 (br s, 1H), 4.46 (d, 1H, *J*=2.4 Hz), 1.82 (s, 3H), 1.81 (s, 3H), 1.64 (s, 3H). MS ESI (pos.) m/e: 599.0 (2M + Na).

**2 (+/-)-3**

**(+/-)-3-(4-Hydroxy-phenyl)-hex-4-ynoic acid (3)**  A 5L RB flask was charged with crude compound **2** in 3-pentanone (1 L) and water (500 mL) was added. The suspension was heated to reflux for 48 hours. After cooling, the aqueous layer was adjusted to pH 9 with 3N KOH. The layers were separated and the organic layer was discarded. The aqueous layer was then acidified to pH < 2 with conc. HCl and extracted with EtOAc (2L). The organic layer was dried over MgSO_4_, filtered, and concentrated to a light brown oil. 2L of hexane was added and the mixture was stirred vigorously until the residue solidified. The solid was then triturated with 5% EtOAc/Hex for 4 hours. The solid was collected and dried. Compound **3** was obtained as an off-white powder (139 g, 681 mmol, 85% for two steps). ^1^H NMR(500MHz, DMSO-*d*_6_) δ ppm  12.2 (s, 1H), 9.27 (s, 1H), 7.12 (d, 2H, *J*=8.5 Hz), 6.67 (d, 2H, *J*=8.6 Hz), 3.87 (m, 1H), 2.54 (m, 2H), 1.82 (d, 3H, *J*=2.4 Hz). MS ESI (pos.) m/e: 205.1 (M + H); 227.1 (M + Na).

**(+/-)-3 (*S*)-3**

**(*3S*)-3-(4-Hydroxy-phenyl)-hex-4-ynoic acid (3)**  A 12 L round-bottom flask was charged with compound **1.3** (139 g, 681 mmol) and 2-propanol (3.3 L) and then heated to 70 °C. (*1S, 2R*)-1-amino-2-indanol (101 g, 681 mmol) was dissolved in 2-propanol (3.2 L) with gentle warming. The solution of amine was added to the dissolved carboxylic acid and the resulting solution was allowed to cool to room temperature. After 16 hours, the crystals were collected and dried. The salt was re-suspended in 6.5 L of 2-propanol and dissolved by heating to reflux. After allowing to cool to room temperature, the salt was collected after 16 hours. A small sample of the salt was decomposed with aqueous acid as described below and the free carboxylic acid was then analyzed by chiral HPLC (Daicel ChiralPAK AD-H column, eluant: 0.1% TFA in 90:10 hexanes:2-propanol) and was found to have 97% ee. The salt was suspended in ethyl acetate (500 mL) and water (500 mL). 2N HCl (400 mL) was added with vigorous mixing. After two clear layers were obtained, the layers were separated and the aqueous layer extracted with ethyl acetate (500 mL). The combined extracts were washed with saturated brine, dried over MgSO_4_, filtered, and concentrated to a light yellow oil which crystallized on drying *in vacuo*. The resolved compound **3** was obtained as an off-white solid (51.2 g, 251 mmol, 37%).

***(S)-*3 4**

***(3S)*-3-(4-Hydroxy-phenyl)-hex-4-ynoic acid methyl ester (4).** Phenol **3** (51.2 g, 251 mmol) was dissolved in methanol (500 mL) and treated with conc. H_2_SO_4_ (5 drops). The reaction stirred at reflux for 14 hours. The solution was cooled to room temperature and concentrated by rotary evaporator. The residue was partitioned between EtOAc (500 mL) and sat. NaHCO_3_(aq) (500 mL). The organic layer was dried over MgSO_4_, filtered, and concentrated to a yellow oil (52.0 g, 238 mmol, 95%). ^1^H NMR(500MHz, acetone-*d*_6_) δ ppm  8.2 (br s, 1H), 7.20 (d, 2H, *J*=9.5 Hz), 6.77 (d, 2H, *J*=9.0 Hz), 3.98 (m, 1H), 3.60 (s, 3H), 2.65 (m, 2H), 1.78 (d, 3H, *J*=2.5 Hz). MS ESI (pos.) m/e: 219.1 (M + H); 241.1 (M + Na).

**5**

**3-(4-Trifluoromethylphenyl)-benzoic acid (5).** The Suzuki coupling was carried out according to the method of Dyer et al. (*Tetrahedron Letters*, **2001**, *42*, 1765-1767). Commercially available 4-(Trifluoromethyl)phenylboronic acid (95.9g, 505 mmol) and 3-bromobenzoic acid (100.5g, 500 mmol) were suspended in 2-propanol:water (1:4, 500 mL). 10% Pd/C (4.8g) was added. Aqueous Na_2_CO_3_ (250 mL, 20% by wt.) was then added slowly (CO_2_ evolution !) to the above mixture while vigorously stirring. The resulting mixture was stirred at room temperature for 30 min and then heated at 70°C for 4 hours. The Pd/C was filtered and rinsed with 20% aqueous Na_2_CO_3_ solution (20mL). The filtrate was acidified to pH~4 with 6N HCl, and the resulting white solid was collected by filtration and dried *in* *vacuo*. The crude product was dissolved in acetone (1.4L) then an equal volume of distilled water was added and the resulting white precipitate was collected by filtration and dried *in vacuo*. 120.2 g of **5** was acquired (yield: 90%). MS ESI m/e: 267 (M-H). ^1^H NMR (500MHz, DMSO-*d*_6_) δ ppm 13.17 (1H, s), 8.25 (1H, s), 8.03-7.99 (2H, m), 7.95 (2H, d), 7.85 (2H, d), 7.66 (1H, t). mp: 197 °C – 200 °C.

**5** **6**

**3-(4-Trifluoromethylphenyl)-benzyl alcohol (6).** Carboxylic acid **5** (123.9g, 466 mmol) in anhydrous THF (800mL) was added dropwise to LiAlH_4_ in THF solution (1M, 520 mL) at 0 °C over 2.5 hours. The resulting mixture was slowly warmed to room temperature and stirred for another 4 hours. The reaction was slowly quenched sequentially with water (25mL) at 0°C, 15% NaOH aqueous solution (25mL) and another portion of water (75mL). The resulting mixture was stirred overnight. The precipitate was filtered and rinsed with acetone (10mL x 3). The filtrate was concentrated to ~150mL and then partitioned between ethyl acetate and water. The combined organic phases were dried over Na_2_SO_4_ and concentrated to give a white solid (114g, 97%). The crude product (**6**) was used in the next step without further purification. MS ESI m/e: 253 (M-H). ^1^H NMR (500MHz, CDCl_3_) δ ppm 7.75-7.71 (4H, m), 7.65 (1H, s),  7.57-7.55 (1H, m), 7.50-7.45 (2H, m), 4.6 (2H, s). mp: 62 °C – 63 °C.

**6** **7**

**3-(4-Trifluoromethylphenyl)-benzyl bromide (7).** The alcohol **6** (52.7g, 400mmol) was dissolved in anhydrous dichloromethane (200mL). Thionyl bromide (1.1 equiv) was slowly added dropwise to the above solution. The resulting mixture was stirred at room temperature for 4 hours. After TLC indicated reaction completion, the organic phase was rinsed with water twice and then dried over Na_2_SO_4_ and concentrated to give a white solid. The crude product was dissolved in hot n-heptane (~1 g/mL) and then cooled in an ice bath for 1 hour before collection of the precipitate by filtration. A second and third crop of the desired bromide could be obtained by concentrating the filtrate and repeating the crystallization. The crops were combined and dried *in vacuo* to give a white solid (60.65 g, 92%). ^1^H NMR (500MHz, CDCl_3_) δ ppm 7.75-7.72 (4H, m), 7.71 (1H, s), 7.65-7.55 (1H, s), 7.50-7.46 (2H, m), 4.60 (2H, s). mp: 62-64 °C.

**4 7 8**

***(3S)-*3-[4-(4'-Trifluoromethyl-biphenyl-3-ylmethoxy)-phenyl]-hex-4-ynoic acid methyl ester (8).** Phenol **4** (38.2 g, 175 mmol) was dissolved in acetone (250 mL) and treated with Cs_2_CO_3_ (62.2 g, 191 mmol). After 10 minutes, benzyl bromide **7** (52.5 g, 166 mmol) was added in one portion. The reaction was stirred at room temperature for 48 h then filtered and concentrated to an pale yellow oil which was purified by filtration through silica gel (1 L of silica gel, elution with 3 L of 15% then 2 L of 20% iso-propyl acetate in heptane). The first 4 L of eluant was concentrated to 79 g of pale yellow oil. Analysis by ^1^H-NMR shows 5% iso-propyl acetate and 1.5% heptane by weight are still present after drying *in vacuo* for 16 hours, so actual yield is 73.8 g (97%). ^1^H NMR (500MHz, DMSO-d_6_) δ ppm 7.91 (2H, d, *J*=8.4 Hz), 7.84 (3H, m), 7.71 (1H, m), 7.55 (2H, m), 7.29 (2H, d, *J*=8.5 Hz), 7.00 (2H, m), 5.19 (2H, s), 3.98 (1H, m), 3.57 (3H, s), 2.71 (2H, d, *J*=8.0 Hz), 1.78 (3H, d, *J*=2.5 Hz). MS ESI m/e: 453 (M+H).

**8 (AMG 837)**

***(3S)-*3-[4-(4'-Trifluoromethyl-biphenyl-3-ylmethoxy)-phenyl]-hex-4-ynoic acid (AMG 837**). Ester **8** (72.8 g, 161 mmol) was dissolved in denatured ethanol (90:5:5 ethanol:methanol:2-propanol, 390 mL) and then treated with 10% NaOH_(aq)_ (130 mL). The milky suspension was stirred at room temperature for 5 hrs, then the resulting clear solution was neutralized with 3 N HCl _(aq.)_  to pH 1-2. The mixture was concentrated under reduced pressure and extracted with 1:1 iso-propyl acetate:hexanes (2 x 400 mL). The combined organic phases were washed with saturated brine, dried over MgSO_4_, filtered and concentrated. Compound **AMG837** was obtained as a pale yellow glass (80g, containing iso-propyl acetate by ^1^H-NMR) ^1^H NMR (500MHz, DMSO-d_6_) δ ppm 12.25 (1H, s), 7.93 (2H, d, *J*=10 Hz), 7.84 (3H, m), 7.71 (1H, d, *J*=10 Hz), 7.55 (2H, m), 7.29 (2H, d, *J*=10 Hz), 7.00 (2H, d, *J*=10 Hz), 5.19 (2H, m), 3.95 (1H, m), 2.61 (2H, m), 1.78 (3H, s). MS ESI m/e: 437 (M-H).

**AM-8596**

**3-(4-((3'-methoxy-4-biphenylyl)methoxy)phenyl)-4-hexynoic acid (AM-8596).** ^1^H NMR (500 MHz, CH_2_Cl_2_) δ ppm 7.60 - 7.64 (2 H, m), 7.50 (2 H, m, *J*=8.3 Hz), 7.33 - 7.38 (1 H, m), 7.28 - 7.33 (2 H, m), 7.17 - 7.22 (1 H, m), 7.12 - 7.15 (1 H, m), 6.92 - 6.97 (2 H, m), 6.90 (1 H, ddd, *J*=8.2, 2.6, 0.7 Hz), 5.10 (2 H, s), 3.98 - 4.08 (1 H, m), 3.85 (3 H, s), 2.75 - 2.84 (1 H, m), 2.67 - 2.75 (1 H, m), 1.79 - 1.86 (3 H, m). MS ESI (neg.) m/e: 398.2 (M-H)^+^.

**9**

**(2'-fluoro-5'-methoxy-[1,1'-biphenyl]-4-yl)methanol (9).** To a round bottom flask containing (4-iodophenyl)methanol (13.5 g, 57.7 mmol) and 2-Fluoro-5-Methoxyphenyl boronic acid (10.0 g, 58.9 mmol) was added a premixed solution (1:1) of 2-Propanol (75.0 ml) and water (75.0 ml). After stirring at room temperature for ~5 minutes, palladium, 10wt. % on activated carbon (0.901 g, 8.46 mmol) and sodium carbonate anhydrous (6.79 g, 64.1 mmol) were added. The reaction was heated to 80^o^C. After 21 hours, the reaction was cooled to room temperature, then filtered through celite (Rinse with ethanol). The filtrate was concentrated then diluted with water and extracted with ethyl acetate (3 × 100mL). The organic solvent was removed under reduced pressure. The residue was then purified by flash chromatography (SiO_2_ gel 60, eluted with 40% EtOAc in hexanes). Fractions containing the desired product were combined and concentrated to a light yellow oil **9** (13.3g, 99%). ^1^H NMR (400 MHz, CDCl_3_) δ ppm 7.53 - 7.59 (2 H, m), 7.45 (2 H, d, *J*=8.4 Hz), 7.08 (1 H, dd, *J*=10.0, 9.0 Hz), 6.95 (1 H, dd, *J*=6.3, 3.1 Hz), 6.84 (1 H, dt, *J*=8.9, 3.4 Hz), 4.74 (2 H, s), 3.83 (3 H, s), 1.99 (1 H, s). MS ESI (pos.) m/e: 250.1 (M+H_2_O)^+^.

**9 10**

**4'-(chloromethyl)-2-fluoro-5-methoxy-1,1'-biphenyl (10).** The alcohol **2** (8.44 g, 36.4 mmol, 1.0 eq., MW 232.26) was dissolved in anhydrous dichloromethane (40 mL). Thionyl chloride (6.5 g, 136 mmol, 1.5 eq., MW 119) was slowly added dropwise to the above solution. The resulting mixture was stirred at room temperature for 16 hours. The organic solvent was removed *in vacuo*. The residue was then purified by flash chromatography (SiO_2_ gel 60, eluted with 10% EtOAc in hexanes). Fractions containing the desired product **10** were combined and concentrated to a colorless solid (8.2 g, 90%). ^1^H NMR (400 MHz, CDCl_3_) δ ppm 7.53 - 7.59 (2 H, m), 7.45 - 7.51 (2 H, m), 7.09 (1 H, dd, *J*=10.0, 9.0 Hz), 6.95 (1 H, dd, *J*=6.3, 3.1 Hz), 6.86 (1 H, dt, *J*=8.9, 3.4 Hz), 4.65 (2 H, s), 3.84 (3 H, s).

**11**

**2'-fluoro-5'-methoxy-[1,1'-biphenyl]-4-carboxylic acid (11).** The Suzuki coupling was carried out according to the method of Dyer *et al*. (*Tetrahedron Letters*, **2001**, *42*, 1765-1767). Commercially available 2-Fluoro-5-Methoxyphenyl boronic acid (10.0 g, 58.8 mmol, 1.05 eq., MW 169.95) and 4-bromobenzoic acid (11.2 g, 55.7 mmol, 1.0 eq., MW 201.02) were suspended in 2-propanol:water (1:1, 100 mL). 10% Pd/C (1.0g, 0.1 eq.) was added followed by Na_2_CO_3_ (5.91 g, 55.7 mmol, 1.0 eq.). The resulting mixture was heated at 80°C for 38 hours. The mixture was filtered through celite® and rinsed with water. The filtrate was acidified to pH=2. The white solid was filtered and dried *in* *vacuo*. The crude material (**11**) (11.3g) was used in the next step without further purification. ^1^H NMR (500 MHz, CDCl_3_) δ ppm 8.20 (2 H, d, *J*=8.3 Hz), 7.67 (2 H, dd, *J*=8.3, 1.5 Hz), 7.09 - 7.15 (1 H, m), 6.98 (1 H, dd, *J*=6.1, 3.2 Hz), 6.90 (1 H, dt, *J*=8.9, 3.4 Hz), 3.85 (3 H, s)

**11 9**

**(2'-fluoro-5'-methoxy-[1,1'-biphenyl]-4-yl)methanol (9).** To the carboxylic acid **11** (10.8 g, 43.7 mmol, 1.0 eq., MW 246.24) in anhydrous THF (200mL) was added dropwise a solution of LiAlH_4_ in THF (1.0 M, 66 mL, 66 mmol, 1.5 eq) at 0°C over 30 minutes. The resulting mixture was slowly warmed to room temperature and stirred for 4 hours. The reaction was slowly quenched with water (10 mL) at 0°C, 1N NaOH aqueous solution (50 mL) and another portion of water (10 mL). The mixture was filtered and the filtrate was extracted with EtOAc (3 × 100 mL), dried over MgSO_4_ and concentrated to give colorless oil (8.44 g, 83%). The crude product (**9**) was used in the next step without further purification. ^1^H NMR (400 MHz, CDCl_3_) δ ppm 7.53 - 7.59 (2 H, m), 7.45 (2 H, d, *J*=8.4 Hz), 7.08 (1 H, dd, *J*=10.0, 9.0 Hz), 6.95 (1 H, dd, *J*=6.3, 3.1 Hz), 6.84 (1 H, dt, *J*=8.9, 3.4 Hz), 4.74 (2 H, s), 3.83 (3 H, s), 1.99 (1 H, s). MS ESI (pos.) m/e: 250.1 (M+H_2_O)^+^.

**9 10**

**4'-(chloromethyl)-2-fluoro-5-methoxy-1,1'-biphenyl (10).** The crude alcohol **9** (8.44 g, 36.4 mmol, 1.0 eq., MW 232.26) was dissolved in anhydrous dichloromethane (40 mL). Thionyl chloride (6.5 g, 136 mmol, 1.5 eq., MW 119) was slowly added dropwise to the above solution. The resulting mixture was stirred at room temperature for 16 hours. The organic solvent was removed *in vacuo*. The residue was then purified by flash chromatography (SiO_2_ gel 60, eluted with 10% EtOAc in hexanes). Fractions containing the desired product **10** were combined and concentrated to a colorless solid (8.2 g, 90%). ^1^H NMR (400 MHz, CDCl_3_) δ ppm 7.53 - 7.59 (2 H, m), 7.45 - 7.51 (2 H, m), 7.09 (1 H, dd, *J*=10.0, 9.0 Hz), 6.95 (1 H, dd, *J*=6.3, 3.1 Hz), 6.86 (1 H, dt, *J*=8.9, 3.4 Hz), 4.65 (2 H, s), 3.84 (3 H, s).

**12 13**

**(3*S*)-ethyl 3-(4-((tetrahydro-2H-pyran-2-yl)oxy)phenyl)pent-4-ynoate (13).** To stirred solution of phenol **12** (1.0g, 4.6 mmol, 1 eq., MW 218.25) in dichloromethane at 23 ^o^C was added 3,4-Dihydro-2H-pyran (839 μL, 9.2 mmol, 2 eq., MW 84.12) followed by PPTS (catalytic, MW 251.31). The resulting mixture was stirred for 16 hours and concentrated *in vacuo*. The residue was then purified by flash chromatography (SiO_2_ gel 60, eluted with 0 to 20% EtOAc in hexanes). Fractions containing the desired product **13** were combined and concentrated to a colorless oil (1.3 g, 94%). MS ESI (pos.) m/e: 325.1 (M+Na)^+^, 320.2 (M+H_2_O)^+^.

**13 14 14.1**

**(*3S,E*)-ethyl 3-(4-((tetrahydro-2H-pyran-2-yl)oxy)phenyl)-5-(tributylstannyl)pent-4-enoate (14).**  To a stirred solution of **13** (80.0 mg, 0.26 mmol, 1eq., MW 302.37) in THF at 23 ^o^C was added PdCl_2_(PPh_3_)_2_ (18.6 mg, 0.026 mmol, 0.1 eq., MW 701.89) followed by Bu_3_SnH (84.0 μL, 0.32 mmol, 1.2 eq., MW 291.05). After the addition the solution turned black. After a further 2 mins, the mixture was concentrated *in vacuo*. The residue was then purified by flash chromatography (SiO_2_ gel 60, eluted with 0 to 20% EtOAc in hexanes). Fractions containing the desired product **14** were combined and concentrated to a colorless oil (96.0 mg). ^1^H NMR spectroscopy shows the product to be a 3:1 ratio of **14**:**14.1** respectively.

**14 15**

**(*3S,E*)-ethyl 5-iodo-3-(4-((tetrahydro-2H-pyran-2-yl)oxy)phenyl)pent-4-enoate (15).**  To a stirred solution of **14/14.1** (96.0 mg, 0.16 mmol, 1eq., MW 593.42) in THF (5 mL) at -78 ^o^C was added iodine (45.0 mg, 0.18 mmol, 1.1 eq., MW 253.81) in THF (2 mL) dropwise. After the addition was complete, a saturated solution of NaS_2_O_3_ and NaHCO_3_ were added at the same time to quench the reaction. EtOAc was added to the mixture and washed with NaHCO_3_ (aq) (2 x 50 mL), brine (1 x 50 mL) and dried with MgSO_4_ and filtered. The organic layer was concentrated *in vacuo*. The residue was then purified by flash chromatography (SiO_2_ gel 60, eluted with 0 to 20% EtOAc in hexanes). Fractions containing the desired product **15** were combined and concentrated to a colorless oil (66 mg, 58% over 2 steps). ^1^H NMR (500 MHz, CDCl_3_) δ ppm 7.08 - 7.13 (2 H, m), 7.01 (2 H, m, *J*=8.6 Hz), 6.66 (1 H, dd, *J*=14.4, 7.6 Hz), 6.10 (1 H, d, *J*=14.4 Hz), 5.39 (1 H, t, *J*=3.2 Hz), 4.04 - 4.16 (2 H, m), 3.87 - 3.94 (1 H, m), 3.84 (1 H, q, *J*=7.5 Hz), 3.56 - 3.64 (1 H, m), 2.61 - 2.77 (2 H, m), 1.94 - 2.07 (1 H, m), 1.82 - 1.89 (2 H, m), 1.57 - 1.74 (4 H, m), 1.20 (3 H, t, *J*=7.1 Hz). MS ESI (pos.) m/e: 453.0 (M+Na)^+^, 448.1 (M+H_2_O)^+^.

**15 16**

**(*3S,E*)-ethyl 3-(4-((tetrahydro-2H-pyran-2-yl)oxy)phenyl)hex-4-enoate (16).** To a stirred solution of **15** (350.0 mg, 0.81 mmol, 1eq., MW 430.29) in THF (20 mL) at 23 ^o^C was added Pd(PPh_3_)_4_ (94 mg, 0.081 mmol, 0.1 eq., MW 1155.58) followed by dropwise addition of Me_2_Zn (976 μL, 0.97 mmol, 1.2 eq., 1.0 M). The yellow color disappears on addition of the Me_2_Zn, after 30 mins the color returns signaling the end of the reaction. Water (10 mL) was added to quench the reaction. The mixture was extracted with EtOAc (2 x 50 mL) dried with MgSO_4_ and filtered. The organic layer was concentrated *in vacuo*. The residue was then purified by flash chromatography (SiO_2_ gel 60, eluted with 0 to 20% EtOAc in hexanes). Fractions containing the desired product **16** were combined and concentrated to a colorless oil (180 mg, 69%). ^1^H NMR (500 MHz, CDCl_3_) δ ppm 7.12 (2 H, m, *J*=8.6 Hz), 6.99 (2 H, m, *J*=8.6 Hz), 5.54 - 5.62 (1 H, m), 5.43 - 5.52 (1 H, m), 5.38 (1 H, t, *J*=3.2 Hz), 4.04 - 4.11 (2 H, m), 3.88 - 3.96 (1 H, m), 3.76 (1 H, q, *J*=7.6 Hz), 3.56 - 3.63 (1 H, m), 2.60 - 2.70 (2 H, m), 1.95 - 2.05 (1 H, m), 1.80 - 1.89 (2 H, m), 1.57 - 1.73 (6 H, m), 1.15 - 1.22 (3 H, m). MS ESI (pos.) m/e: 341.2 (M+Na)^+^, 336.2 (M+H_2_O)^+^. The desired product was contaminated (~5%) with a further olefinic product (believed to contain a terminal double bond).

**16 17**

**(*S,E*)-ethyl 3-(4-hydroxyphenyl)hex-4-enoate 17.** To a stirred solution of **16** (180.0 mg, 0.57 mmol, MW 318.42) in EtOH (5 mL) at 23 ^o^C was added PPTS (catalytic), stirring continued for 16 h. The reaction was concentrated *in vacuo*. The residue was then purified by flash chromatography (SiO_2_ gel 60, eluted with 0 to 20% EtOAc in hexanes). Fractions containing the desired product **6** were combined and concentrated to a colorless oil. The desired product was contaminated (~5%) with a further olefinic product (believed to contain a terminal double bond). This impurity was removed by further purification on silica gel containing 10% AgNO_3_ eluting with 0 to 20% EtOAc in hexanes. The combined fractions were concentrated under reduced pressure to afford **17** (120 mg, 91%) as a colorless oil. ^1^H NMR (500 MHz, CDCl_3_) δ ppm 7.07 (2 H, m, *J*=8.3 Hz), 6.73 - 6.78 (2 H, m), 5.53 - 5.61 (1 H, m), 5.43 - 5.51 (1 H, m), 4.97 (1 H, br. s.), 4.01 - 4.12 (2 H, m), 3.69 - 3.80 (1 H, m), 2.58 - 2.72 (2 H, m), 1.66 (3 H, d, *J*=6.1 Hz), 1.13 - 1.22 (3 H, t, *J*=7.1 Hz). MS ESI (pos.) m/e: 252.1 (M+H_2_O)^+^, 235.1 (M+H)^+^,

**17 18**

**(*S,E*)-ethyl 3-(4-((2'-fluoro-5'-methoxy-[1,1'-biphenyl]-4-yl)methoxy)phenyl)hex-4-enoate (18).** To a stirred solution of **17** (17.0 mg, 0.073 mmol, 1eq., MW 234.30) in DMF (2 mL) at 23 ^o^C was added **10** (21.8 mg, 0.087 mmol, 1.2 eq., MW 250.70) followed by Cs_2_CO_3_ (28.4 mg, 0.087 mmol, 1.2 eq., MW 325.82). The reaction mixture was further stirred for 16h, then diluted with ethyl acetate (30 mL), washed with water and brine, dried over MgSO_4_ and filtered and concentrated *in vacuo*. The residue was then purified by flash chromatography (SiO_2_ gel 60, eluted with 0 to 20% EtOAc in hexanes). Fractions containing the desired product **18** were combined and concentrated to a colorless oil (28.0 mg, 86%). ^1^H NMR (500 MHz, CDCl_3_) δ ppm 7.55 - 7.59 (2 H, m), 7.48 - 7.53 (2 H, m), 7.15 (2 H, d, *J*=8.6 Hz), 7.05 - 7.11 (1 H, m), 6.91 - 6.97 (3 H, m), 6.84 (1 H, dt, *J*=8.9, 3.4 Hz), 5.53 - 5.63 (1 H, m), 5.43 - 5.53 (1 H, m), 5.09 (2 H, s), 4.04 - 4.11 (2 H, m), 3.83 (3 H, s), 3.77 (1 H, q, *J*=7.6 Hz), 2.61 - 2.71 (2 H, m), 1.66 (3 H, d, *J*=6.4 Hz), 1.18 (3 H, t, *J*=7.1 Hz). MS ESI (pos.) m/e: 471.1 (M+Na)^+^, 466.1 (M+H_2_O)^+^.

**18 AM-8182**

**(*S,E*)-3-(4-((2'-fluoro-5'-methoxy-[1,1'-biphenyl]-4-yl)methoxy)phenyl)hex-4-enoic acid AM-8182**. To a stirred solution of **7** (28.0 mg, 0.06 mmol, 1 eq., MW 448.54) in THF/EtOH (2 mL, 3/1, v/v) was added 1N NaOH (aq) (1 mL). The reaction mixture was stirred at 23 ^o^C for 16 hours. 1N HCl was added to acidify the mixture to pH 2. The mixture was extracted with ethyl acetate (2 x 10 mL), concentrated *in vacuo*. The residue was then purified by flash chromatography (SiO_2_ gel 60, eluted with 0 to 30% EtOAc in hexanes). Fractions containing the desired product **AM-8182** were combined and concentrated to a colorless oil (21.8 mg, 83%). ^1^H NMR (500 MHz, CDCl_3_) δ ppm 7.55 - 7.59 (2 H, m), 7.48 - 7.53 (2 H, m), 7.15 (2 H, d, *J*=8.6 Hz), 7.08 (1 H, t, *J*=9.4 Hz), 6.92 - 6.97 (3 H, m), 6.84 (1 H, dt, *J*=9.0, 3.4 Hz), 5.55 - 5.63 (1 H, m), 5.46 - 5.55 (1 H, m), 5.09 (2 H, s), 3.83 (3 H, s), 3.78 (1 H, q, *J*=7.3 Hz), 2.67 - 2.77 (2 H, m), 1.67 (3 H, d, *J*=6.1 Hz). MS ESI (neg.) m/e: 839 (2M-H)^+^, 419.2 (M-H)^+^.

**19**

**5,5-Dimethylcyclopent-1-enyl trifluoromethanesulfonate (19).** To a solution of 2,2-dimethylcyclopentanone (available from ChemSampCo) (10.00g, 89 mmol) in THF (100 mL), was slowly added LDA (49 ml, 98 mmol, 2.0 M, in heptane) at -78 ºC. The resulting mixture was stirred at -78 ºC for 1 hour. A solution of N-phenyltriflimide (33 g, 94 mmol) in THF (100 ml) was added to the mixture at -78 ºC, and stirring was continued at 0 ºC for 2 hours and then at room temperature overnight. The reaction mixture was extracted with hexane (80× 2 mL). The organic layer was washed with saturated Na_2_CO_3_ (30 mL), brine (20 mL), and dried with MgSO_4_. The organic phase was concentrated in vacuo, the residue was then purified by flash chromatography (SiO_2_ gel 60, eluted with 0%–10% EtOAc in hexanes). Fractions containing the desired product were combined and concentrated to provide **19** (10.05g, 46%). ^1^H NMR (500 MHz, CDCl_3_) δ ppm 5.55 (1 H, t, *J*=2.6 Hz), 2.36 (2 H, td, *J*=7.1, 2.7 Hz), 1.82 - 1.88 (2 H, m), 1.13 - 1.17 (6 H, s).

**19 20**

**2-(5,5-Dimethylcyclopent-1-enyl)-4,4,5,5-tetramethyl-1,3,2-dioxaborolane (20).** PdCl_2_(PPh_3_)_2_ (1.82 g, 2.59 mmol), PPh_3_ (13.6 g, 51.8 mmol), bis(pinacolato)diboron (15.8 g, 62.1 mmol) and KOPh (fine powder, 9.58 g, 72.5 mmol) were added to a flask. The flask was flushed with nitrogen and charged with toluene (200 mL) and with **19** (12.64 g, 51.8 mmol). The mixture was stirred at 50 ºC for 2 hours. The reaction mixture was treated with water at room temperature and extracted with benzene (60 × 2 mL). The organic layer was dried over MgSO_4_ and concentated in vacuo. The residue was then purified by flash chromatography (SiO_2_ gel 60, eluted with 0%-10% EtOAc in hexanes). Fractions containing the desired product were combined and concentrated to provide **20** (6.7, 58%). ^1^H NMR (400 MHz, CDCl_3_) δ ppm 6.38 (1 H, t, *J*=2.3 Hz), 2.37 (2 H, td, *J*=7.3, 2.3 Hz), 1.65 (2 H, t, *J*=7.3 Hz), 1.24 - 1.29 (12 H, s), 1.11 - 1.15 (6 H, s).

**21**

**Methyl 3-bromo-4-hydroxybenzoate (21).** To a stirred solution of 3-bromo-4-hydroxybenzoic acid (available from Alfa Aesar, Avocado, Lancaster) (50.0 g, 231 mmol) in MeOH (300 mL) was added a cold solution of sulfuric acid (2.50 mL, 47 mmol). The mixture was heated to 80 °C and monitored by TLC. After 16.5 hours, the solvent was removed and the reaction mixture was diluted with EtOAc. The organic phase was washed carefully two times with saturated aqueous NaHCO_3_, once with brine, and then dried over anhydrous sodium sulfate. After filtration, the organic solvent was removed in vacuo to yield **21** as a white solid (yield 100%) that was used without purification. ^1^H NMR (400 MHz, DMSO*-d*_6_) δ ppm 11.26 (1 H, br. s.), 8.01 (1 H, d, *J*=2.0 Hz), 7.79 (1 H, dd, *J*=8.4, 2.2 Hz), 7.04 (1 H, d, *J*=8.4 Hz), 3.80 (3H, s). ESI (pos.) m/e: 232.9, 230.9 (M+H)^+^.

**21 22**

**Methyl 3-bromo-4-(tetrahydro-2H-pyran-2-yloxy)benzoate (22).** To a stirred solution of **21** (38 g, 164 mmol) and 3,4-dihydro-2H-pyran (45 mL, 493 mmol) in CH_2_Cl_2_ (355 mL) was added 4-methylbenzenesulfonic acid hydrate (0.63 g, 3.30 mmol). The mixture was stirred at room temperature and monitored by TLC. After 2 hours, the solution was washed with a mixed aqueous solution of saturated aqueous sodium bicarbonate/brine/water (1:1:2). The aqueous layer was extracted three times with ether. After drying over anhydrous sodium sulfate and then filtering, the organic solvent was removed under reduced pressure. The residue was then purified by flash chromatography (SiO_2_ gel 60, eluted with 0%–10% EtOAc in hexanes). Fractions containing the desired product were combined and concentrated to provide a white solid. The product was recrystallized from MeOH to provide **22** (yield 90%). ^1^H NMR (400 MHz, CDCl_3_) δ ppm 7.79 (1 H, d, *J*=1.8 Hz), 7.62 (1 H, d, *J*=8.2 Hz), 7.55 (1 H, dd, *J*=8.3, 1.9 Hz), 5.63 (1 H, t, *J*=2.7 Hz), 3.91 (3 H, s), 3.83 - 3.89 (1 H, m), 3.61 - 3.68 (1 H, m), 2.06 - 2.20 (1 H, m), 1.96 - 2.04 (1 H, m), 1.84 - 1.96 (1 H, m), 1.68 - 1.81 (2 H, m), 1.60 - 1.68 (1 H, m). ESI (pos.) m/e: 337.0, 339.0 (M+Na)^+^.

**22 23**

**Methyl 3-(5,5-dimethylcyclopent-1-enyl)-4-(tetrahydro-2H-pyran-2-yloxy)benzoate (23).** A stirred mixture of **22** (10.1 g, 31.9 mmol), ground S-Phos (2.62 g, 6.39 mmol), palladium aceate (0.72 g, 3.2 mmol), and potassium phosphate, tribasic (17.0 g, 80.2 mmol) in DMF (70 mL) and water (3.5 mL) was purged three times with argon and placed under vacuum three times. Before heating, 2-(5,5-dimethylcyclopent-1-enyl)-4,4,5,5-tetramethyl-1,3,2-dioxaborolane (**20**) (8.50 g, 38.3 mmol) was added *via* syringe. The resulting mixture was then heated to 75 °C. After 21 hours (black solution), the reaction was cooled to room temperature, diluted with water, and extracted three times with EtOAc. The organic layers were combined and washed twice with brine. After drying over anhydrous sodium sulfate and filtering, the organic solvent was removed under reduced pressure. The residue was then purified by flash chromatography (SiO_2_ gel 60, eluted with 0%–20% EtOAc in hexanes). Fractions containing the desired product were combined and concentrated to provide **23** as a colorless oil that solidified on standing (yield 80%). ^1^H NMR (400 MHz, CDCl_3_) δ 7.91 (1 H, dd, *J*=8.6, 2.3 Hz), 7.74 (1 H, d, *J*=2.3 Hz), 7.15 (1 H, d, *J*=8.6 Hz), 5.55 (1 H, t, *J*=2.3 Hz), 5.49 (1 H, t, *J*=2.9 Hz), 3.88 (3 H, s), 3.82 (1 H, td, *J*=11.1, 2.9 Hz), 3.64 (1 H, m), 2.43 (2 H, td, *J*=7.0, 2.3 Hz), 1.77-1.95 (5 H, m), 1.69 (1 H, m), 1.61 (2 H, m), 1.11 (3 H, s), 1.07 (3 H, s).. ESI (pos.) m/e: 331.1 (M+H)^+^.

**23 24**

**Methyl 3-(5,5-dimethylcyclopent-1-enyl)-4-hydroxybenzoate (24).** To a stirred solution of **23** (19.0 g, 57.6 mmol) in MeOH (150 mL) was added pyridinium para-toluenesulfonate (PPTS) (1.46 g, 5.80 mmol). The mixture was heated to 50 °C and monitored with TLC. After 19 hours, the organic solvent was removed under reduced pressure and the residue was then purified by flash chromatography (SiO_2_ gel 60, eluted with 0%–15% EtOAc in hexanes). Fractions containing the desired product were combined and concentrated to provide **24** as a white solid (yield 90%). ^1^H NMR (400 MHz, CDCl_3_) δ 7.89 (1 H, dd, *J*=8.6, 2.0 Hz), 7.79 (1 H, d, *J*=2.3 Hz), 6.97 (1 H, d, *J*=8.6 Hz), 5.87 (1 H, s), 5.81 (1 H, t, *J*=2.3 Hz), 3.89 (3 H, s), 2.51 (2 H, td, *J*=7.1, 2.5 Hz), 1.94 (2 H, t, *J*=7.0 Hz), 1.12 (6 H, s). ESI (pos.) m/e: 247.1 (M+H)^+^.

**24 25**

**Methyl 3-(5,5-dimethylcyclopent-1-enyl)-4-(trifluoromethylsulfonyloxy)benzoate (25).** To a stirred solution of **24** (6.00 g, 24.4 mmol) in dry DCM (35 mL) was added TEA (6.80 mL, 48.9 mmol) and 4-dimethylaminopyridine (0.30 g, 2.5 mmol). After about 20 minutes, N-phenyl bis-trifluoromethane sulfonimide (10.5 g, 29.3 mmol) was added in portion. Upon complete addition, the solution was stirred at room temperature and monitored with TLC. After 3 hours, the reaction was diluted with brine and extracted three times with DCM. After drying over anhydrous magnesium sulfate and filtering, the organic solvent was removed under reduced pressure and the residue was then purified by flash chromatography (SiO_2_ gel 60, eluted with 0%–10% EtOAc in hexanes). Fractions containing the desired product were combined and concentrated to provide **25** as a colorless oil (yield 88%). ^1^H NMR (400 MHz, CDCl_3_) δ 8.02 (1 H, dd, *J*=8.6, 2.0 Hz), 7.94 (1 H, d, *J*=2.0 Hz), 7.35 (1 H, d, *J*=8.6 Hz), 5.80 (1 H, t, *J*=2.5 Hz), 3.94 (3 H, s), 2.48 (2 H, td, *J*=7.0, 2.3 Hz), 1.91 (2 H, t, *J*=7.0 Hz), 1.09 (6 H, s). ESI (pos.) m/e: 379.0 (M+H)^+^.

**25 26**

**Methyl 2-(5,5-dimethylcyclopent-1-en-1-yl)-2'-fluoro-5'-methoxy-[1,1'-biphenyl]-4-carboxylate (26).** To a stirred solution of **25** (8.71 g, 23.0 mmol) in DMF (20 mL) at 23 °C was added 2-fluoro-5-methoxyphenylboronic acid (7.84 g, 46.1 mmol) and potassium carbonate (9.56 g, 69.1 mmol) followed by tetrakis(triphenylphosphine)palladium (0) (2.67 g, 2.31 mmol). The mixture was heated to 90 C. After 15 hours, LCMS-showed that the reaction was complete. The mixture was then cooled to room temperature and then diluted with water. After extracting three times with EtOAc, the mixture was concentrated in vacuo. The residue was then purified by flash chromatography (SiO_2_ gel 60, eluted with 0%–10% EtOAc in hexanes). Fractions containing the desired product were combined and concentrated to provide **26** as a clear oil that solidified (yield 91%). ^1^H NMR (400 MHz, CDCl_3_) δ 7.98 (1 H, dd, *J*=8.0, 1.8 Hz), 7.91 (1 H, d, *J*=2.0 Hz), 7.40 (1 H, d, *J*=7.8 Hz), 6.98 (1 H, t, *J*=8.8 Hz), 6.85 (2 H, m), 5.55 (1 H, s), 3.95 (3 H, s), 3.77 (3 H, s), 2.27 (2 H, td, *J*=7.0, 2.7 Hz), 1.68 (2 H, t, *J*=7.0 Hz), 0.87 (6 H, s). ESI (pos.) m/e: 355.1 (M+H)^+^.

**26 27**

**(2-(5,5-Dimethyl-1-cyclopenten-1-yl)-2'-fluoro-5'-(methyloxy)-1,1'-biphenyl-4-yl)methanol (27)** To a solution of **26** (1.27 g, 3.6 mmol) in THF (13.0 mL), was slowly added lithium aluminium hydride, (1.0M solution in diethyl ether, 7.2 mL, 7.2 mmol) at 0 °C. After the addition, the reaction mixture was stirred at 40 °C for 1.5 hours, and then at room temperature for 2 hours. A mixture of water (0.22 mL) in THF (2.0 mL) was slowly added and then 15% sodium hydroxide (0.22 mL) was added at 0 °C. Finally, water (0.65 mL) was added at room temperature. The solid was removed by filtration, and the solvent was removed under reduced pressure. The residue was then purified by flash chromatography (SiO_2_ gel 60, eluted with 10%–30% EtOAc in hexanes). Fractions containing the desired product were combined and concentrated to provide **27** (0.96g, 82%). ^1^H NMR (500 MHz, DMSO*-d*_6_) δ ppm 7.28 (1 H, d, *J*=7.8 Hz), 7.22 (1 H, d, *J*=7.8 Hz), 7.17 (1 H, s), 7.09 (1 H, t, *J*=9.2 Hz), 6.87 (1 H, dt, *J*=8.9, 3.6 Hz), 6.81 (1 H, dd, *J*=5.9, 3.2 Hz), 5.48 (1 H, s), 5.24 (1 H, t, *J*=5.7 Hz), 4.54 (2 H, d, *J*=5.9 Hz), 3.34 (1 H, s), 2.19 (2 H, td, *J*=7.0, 2.2 Hz), 1.59 (2 H, t, *J*=7.0 Hz), 0.81 (6 H, s). ESI (pos.) m/e: 309.1 (M-HO)^+^, 345.2 (M+H_2_O)^+^.

**27 28**

**4-(Chloromethyl)-2-(5,5-dimethyl-1-cyclopenten-1-yl)-2'-fluoro-5'-(methyloxy)-1,1'-biphenyl (29)** To a solution of compound **27** (1.10 g, 3.37 mmol) and a catalytic amount of DMF (0.10 mL) in CH_2_Cl_2_ (12.0 mL), was slowly added thionyl chloride (0.802 g, 6.74 mmol) at 0 °C. After addition, the reaction mixture was stirred at room temperature for 1 hour. The solvent was removed under reduced pressure. The residue was then purified by flash chromatography (SiO_2_ gel 60, eluted with 0%–5% EtOAc in hexanes). Fractions containing the desired product were combined and concentrated to provide **28** (1.15g, 99%). ^1^H NMR (400 MHz, CDCl_3_) δ ppm 7.30 - 7.38 (2 H, m), 7.27 (1 H, s), 6.94 - 7.01 (1 H, m), 6.78 - 6.83 (2 H, m), 5.51 - 5.58 (1 H, m), 4.64 (2 H, s), 3.76 (3 H, s), 2.27 (2 H, td, *J*=7.0, 2.5 Hz), 1.63 - 1.72 (2 H, m), 0.83 - 0.92 (6 H, s). ESI (pos.) m/e: 362.2 (M+H_2_O)^+^.

**29**

**(*E*)-methyl 3-cyclopropylacrylate (29).** To a suspension of lithium chloride (22.7 g, 535 mmol) in MeCN (20 mL) was added trimethyl phosphonoacetate (61.7 ml, 428 mmol) and dbu (64.0 ml, 428 mmol), and cyclopropanecarboxaldehyde (26.7 ml, 357 mmol) at 0 °C. The pale yellow mixture was stirred overnight. The mixture was partitioned between water and EtOAc. The layers were separated, and the aqueous phase was extracted with additional EtOAc. The combined organics were washed with 1 N HCl and brine, dried Na_2_SO_4_, and concentrated in vacuo. The crude product was purified by distillation (head 90 °C, bath 120 °C, pressure 30 torr) to afford (*E*)-methyl 3-cyclopropylacrylate **29** as a colorless oil (38.0g, 85%). ^1^H NMR (400 MHz, CDCl_3_) δ ppm 6.43 (1 H, dd, *J*=15.5, 10.1 Hz), 5.90 (1 H, d, *J*=15.5 Hz), 3.72 (3 H, s), 1.51 - 1.65 (1 H, m), 0.91 - 0.99 (2 H, m), 0.60 - 0.68 (2 H, m).

**29** **30**

**(*S*)-methyl 3-cyclopropyl-3-(3-hydroxyphenyl)propanoate (30).** Dioxane/water : 10/1 (2.2 ml) was added to a flask charged with hydroxy[(*S*)-binap]rhodium(I) dimer (29 mg, 20 µmol), and 3-hydroxyphenylboronic acid (273 mg, 1982 µmol) and flushed with nitrogen and (*E*)-methyl 3-cyclopropylacrylate **29** (50 mg, 396 µmol). The resulting mixture was then stirred at 40 °C for 3 h. After evaporation of the solvent, the residue was dissolved in ethyl acetate. The solution was washed with water, brine,and dried over anhydrous Na_2_SO_4_. The residue was then purified by flash chromatography (SiO_2_ gel 60, eluted with 0%–15% EtOAc in hexanes). Fractions containing the desired product were combined and concentrated to provide (*S*)-methyl 3-cyclopropyl-3-(3-hydroxyphenyl)propanoate **30** (72.8 mg, 83% yield) as a colorless oil. ^1^H NMR (400 MHz, CDCl_3_) δ ppm 7.18 (1 H, t, *J*=7.8 Hz), 6.81 (1 H, d, *J*=7.6 Hz), 6.66 - 6.74 (2 H, m), 4.71 (1 H, s), 3.62 (3 H, s), 2.67 - 2.81 (2 H, m), 2.33 (1 H, dt, *J*=9.7, 7.6 Hz), 0.95 - 1.08 (1 H, m), 0.53 - 0.64 (1 H, m), 0.39 - 0.49 (1 H, m), 0.26 (1 H, dq, *J*=9.6, 4.8 Hz), 0.16 (1 H, td, *J*=9.7, 5.0 Hz). MS ESI (pos.) m/e: 238.1 (M+H_2_O)^+^.

**28 30 31**

**(*S*)-methyl 3-cyclopropyl-3-(3-((2-(5,5-dimethylcyclopent-1-en-1-yl)-2'-fluoro-5'-methoxy-[1,1'-biphenyl]-4-yl)methoxy)phenyl)propanoate (31).** A mixture of (*S*)-methyl 3-cyclopropyl-3-(3-hydroxyphenyl)propanoate **30** (0.074 g, 0.33 mmol), **28** (0.11 g, 0.32 mmol), and cesium carbonate (0.16 g, 0.48 mmol) in DMF (1.5 ml) was stirred overnight at room temperature. The mixture was diluted with EtOAc, washed with water and brine, dried (MgSO_4_), and concentrated. The residue was then purified by flash chromatography (SiO_2_ gel 60, eluted with 0%–15% EtOAc in hexanes). Fractions containing the desired product were combined and concentrated to provide **31** as a colorless oil. ^1^H NMR (500 MHz, CDCl_3_) δ ppm 7.41 (1 H, dd, *J*=7.8, 1.7 Hz), 7.35 (1 H, d, *J*=7.8 Hz), 7.31 (1 H, d, *J*=1.5 Hz), 7.25 (1 H, t, *J*=7.8 Hz), 6.94 - 7.00 (1 H, m), 6.84 - 6.92 (3 H, m), 6.77 - 6.82 (2 H, m), 5.53 (1 H, s), 5.10 (2 H, s), 3.76 (3 H, s), 3.63 (3 H, s), 2.69 - 2.82 (2 H, m), 2.37 (1 H, dt, *J*=9.5, 7.6 Hz), 2.26 (2 H, td, *J*=7.0, 2.3 Hz), 1.67 (2 H, t, *J*=7.0 Hz), 0.96 - 1.08 (1 H, m), 0.81 - 0.91 (6 H, s), 0.54 - 0.62 (1 H, m), 0.39 - 0.47 (1 H, m), 0.27 (1 H, dq, *J*=9.6, 4.8 Hz), 0.16 (1 H, dq, *J*=9.7, 4.9 Hz). MS ESI (pos.) m/e: 546.3 (M+H_2_O)^+^.

**AM-1638**

**(3*S*)-3-Cyclopropyl-3-(3-(((2-(5,5-dimethyl-1-cyclopenten-1-yl)-2'-fluoro-5'-(methyloxy)-1,1'-biphenyl-4-yl)methyl)oxy)phenyl)propanoic acid (AM-1638)**. To a solution of **31** (0.15 g, 0.28 mmol) in 2:1 THF/MeOH (4.5 ml) was added 1 N aq. LiOH (1.5 ml, 1.5 mmol). The mixture was stirred overnight at room temperature, quenched with a slight excess of 1 N aq. HCl, and extracted with EtOAc. The combined organics were dried (MgSO_4_) and concentrated. The crude product was purified by silica gel flash chromatography (5–25% EtOAc/hexane) and HPLC (60–90% acetonitrile/water) to afford after lyophilization **AM-1638** as a white foam. ^1^H NMR (400 MHz, CDCl_3_) δ ppm 7.40 (1 H, dd, *J*=7.8, 1.8 Hz), 7.34 (1 H, d, *J*=7.8 Hz), 7.22 - 7.31 (2 H, m), 6.93 - 7.02 (1 H, m), 6.84 - 6.91 (3 H, m), 6.75 - 6.82 (2 H, m), 5.53 (1 H, s), 5.10 (2 H, s), 3.76 (3 H, s), 2.73 - 2.86 (2 H, m), 2.33 - 2.42 (1 H, m), 2.25 (2 H, td, *J*=7.0, 2.4 Hz), 1.66 (2 H, t, *J*=7.0 Hz), 0.98 - 1.09 (1 H, m), 0.85 (6 H, s), 0.60 (1 H, tt, *J*=9.0, 4.5 Hz), 0.40 - 0.48 (1 H, m), 0.31 (1 H, dq, *J*=9.6, 4.8 Hz), 0.17 (1 H, dq, *J*=9.7, 5.0 Hz). ESI (neg.) m/e: 513.3 (M-H)^+^.

**26 32**

**(+/-)-Methyl 2-(2,2-dimethylcyclopentyl)-2'-fluoro-5'-methoxy-[1,1'-biphenyl]-4-carboxylate (32).**  To a stirred solution of **26** (0.660 g, 1.86 mmol) in MeOH (20.00 mL, 1.86 mmol) at 23°C was added Pd/C (0.0198 g, 0.186 mmol). Stirring continued under an atmosphere of hydrogen (0.00375 g, 1.86 mmol) for 16 hours. The reaction mixture was then filtered and concentrated in vacuo to give **32** as a clear oil (0.60 g, 90% yield). ). ^1^H NMR (400 MHz, *CHLOROFORM-d*) δ ppm 8.05 - 8.13 (1 H, m), 7.86 - 7.95 (1 H, m), 7.22 - 7.32 (1 H, m), 6.99 - 7.12 (1 H, m), 6.84 - 6.92 (1 H, m), 6.77 (0.6 H, dd, *J*=5.8, 3.2 Hz), 6.66 (0.4 H, dd, *J*=5.9, 3.1 Hz), 3.95 (3 H, s), 3.81 (3 H, s), 2.99 (0.4 H, t, *J*=9.5 Hz), 2.86 (0.6 H, ddd, *J*=10.4, 8.3, 2.2 Hz), 2.01 - 2.23 (2 H, m), 1.78 - 1.91 (1 H, m), 1.61 - 1.76 (1 H, m), 1.52 - 1.61 (1 H, m), 1.33 - 1.44 (1 H, m), 0.70 - 0.79 (3 H, m), 0.55 - 0.65 (3 H, m). ESI (pos.) m/e: 374.2 (M+H_2_O)^+^, 357.1 (M+H)^+^. ^1^H NMR shows a mixture of compounds (6:4 ratio) with different peaks in the absence of a chiral shift reagent. Without being bound to theory, it is hypothesized that restricted rotation around the biphenyl ring system due to the sterically encumbering dimethylcyclopentane substituent may lead to atropisomers.

**32 33**

**33 33.1 and 33.2**

**(*S*)-(2-(2,2-dimethylcyclopentyl)-2'-fluoro-5'-methoxy-[1,1'-biphenyl]-4-yl)methanol (33.1) and (*R*)-(2-(2,2-dimethylcyclopentyl)-2'-fluoro-5'-methoxy-[1,1'-biphenyl]-4-yl)methanol (33.2).**  To a stirred solution of **32** (0.500 g, 1.4 mmol) in THF (7.0 mL, 1.4 mmol) at 0°C was added LAH (1.4 mL, 1.4 mmol). After addition, the reaction was stirred for 1.5 hours. 1N NaOH (aq) was then added to quench the reaction, and the mixture was then extracted with EtOAc. The organic layers were dried over magnesium sulfate, filtered, and concentrated in vacuo. The resulting product was then purified on silica gel (0%-20% EtOAc/hexane) to give **33** (0.442 g, 96% yield). Chiral separation of **33** was accomplished on Chiracel-OD (3%IPA in hexane) to provide **33.1** and **33.2**. Analytical column (Chiracel-OD (2%IPA in hexane, 45 min run) Peak 1-15.5 mins, Peak 2-38.0 mins). ^1^H NMR (400 MHz, CDCl_3_) δ ppm 7.36 - 7.43 (1 H, m), 7.16 - 7.30 (2 H, m), 6.98 - 7.09 (1 H, m), 6.82 - 6.88 (1 H, m), 6.78 (0.6 H, dd, *J*=5.9, 3.1 Hz), 6.67 (0.4 H, dd, *J*=6.0, 3.2 Hz), 4.75 (2 H, d, *J*=5.9 Hz), 3.80 (3 H, s), 2.98 (0.4 H, t, *J*=9.5 Hz), 2.84 (0.6 H, ddd, *J*=10.5, 8.3, 2.2 Hz), 2.09 - 2.20 (1 H, m), 1.97 - 2.09 (1 H, m), 1.74 - 1.87 (1 H, m), 1.61 - 1.74 (2 H, m), 1.49 - 1.59 (1 H, m), 1.33 - 1.42 (1 H, m), 0.70 - 0.78 (3 H, m), 0.54 - 0.64 (3 H, m). ESI (pos.) m/e: 346.1 (M+H_2_O)^+^. ^1^H NMR shows a mixture of compounds (6:4 ratio) with different peaks in the absence of a chiral shift reagent. Without being bound to theory, it is hypothesized that restricted rotation around the biphenyl ring system due to the sterically encumbering dimethylcyclopentane substituent may lead to atropisomers.

**33.2 34**

**(*R*)-4-(chloromethyl)-2-(2,2-dimethylcyclopentyl)-2'-fluoro-5'-methoxy-1,1'-biphenyl (34).** Thionyl chloride (1.5 mL, 20 mmol) was added to a stirred solution of **33.2** (3.280 g, 10.0 mmol) in DCM (100 mL, 10.0 mmol) and DMF (0.77 mL, 10.0 mmol) at 0°C. Stirring was continued at room temperature for 2 hours. The reaction mixture was then concentrated in vacuo and purified on silica gel (0-10% EtOAc in hexane) to give the desired product **34** (3.00 g, 87 % yield) as a clear oil. ^1^H NMR (400 MHz, CDCl_3_) δ ppm 7.37 - 7.45 (1 H, m), 7.23 - 7.30 (1 H, m), 7.15 - 7.23 (1 H, m), 6.97 - 7.09 (1 H, m), 6.85 (1 H, dt, *J*=8.8, 3.6 Hz), 6.77 (0.6 H, dd, *J*=5.8, 3.2 Hz), 6.67 (0.4 H, dd, *J*=6.0, 3.2 Hz), 4.59 - 4.68 (2 H, m), 3.76 - 3.82 (3 H, m), 2.97 (0.4 H, dd, *J*=10.4, 8.6 Hz), 2.84 (0.6 H, ddd, *J*=10.5, 8.3, 2.2 Hz), 2.10 - 2.23 (0.6 H, m), 1.93 - 2.10 (1.4 H, m), 1.74 - 1.88 (1 H, m), 1.60 - 1.74 (1 H, m), 1.48 - 1.60 (1 H, m), 1.31 - 1.43 (1 H, m), 0.68 - 0.79 (3 H, m), 0.52 - 0.65 (3 H, m). ^1^H NMR shows a mixture of compounds (6:4 ratio) with different peaks in the absence of a chiral shift reagent. Without being bound to theory, it is hypothesized that restricted rotation around the biphenyl ring system due to the sterically encumbering dimethylcyclopentane substituent may lead to atropisomers.

**24 35**

**(*R*)-methyl 3-(2,2-dimethylcyclopentyl)-4-hydroxybenzoate (35).** To a mixture of Rh(cod)_2_BF_4_(Stern Chemical, 35138-22-8, 137.2 mg, 0.338 mmol)/(*R*)-1-[(*S*)-2-(*R*)-(Ditertbutylphosphino)ferrocenyl]ethyl-bis-(3,5-bistrifluoromethylphenyl)phosphine (Solvias, SL-J210-1, 302 mg, 0.3718 mmol), was stirred in THF (300 mL) under N_2_ for 60 minutes and a dark red solution was formed. The resulting solution was added methyl 3-(5,5-dimethylcyclopent-1-enyl)-4-hydroxybenzoate **24** (41.64g, 168.98 mmol) and filled with H_2_ (200psi) three times and stirred at room temperature/200psi for 2h. Passed through a short plug of silica gel, eluting with 1:1 hexane/EtOAc, followed by concentration afforded the desired product as a white solid (98.9A% conversion, 99% yield (41.6 g), 99% ee). ^1^H NMR (400 MHz, CDCl_3_) δ ppm 7.91 (1 H, d, *J*=2.0 Hz), 7.78 (1 H, dd, *J*=8.4, 2.0 Hz), 6.84 (1 H, d, *J*=8.4 Hz), 5.90 (1 H, s), 3.90 (3 H, s), 3.19 (1 H, dd, *J*=10.1, 8.1 Hz), 2.04 - 2.17 (1 H, m), 1.92 - 2.04 (1 H, m), 1.81 - 1.92 (1 H, m), 1.69 - 1.81 (1 H, m), 1.55 - 1.69 (2 H, m), 1.05 (3 H, s), 0.72 (3 H, s). ESI (pos.) m/e: 249.1 (M+H)^+^.

**35 36**

**(*R*)-methyl 3-(2,2-dimethylcyclopentyl)-4-(trifluoromethylsulfonyloxy)benzoate (36).** To a stirred solution of (*R*)-methyl 3-(2,2-dimethylcyclopentyl)-4-hydroxybenzoate (**35**) (18.00 g, 72 mmol) in dichloromethane (181 ml, 72 mmol) at 23^o^C was added triethylamine (12 ml, 87 mmol) and DMAP (catalytic). Followed by n-phenyltriflimide (28 g, 80 mmol), stirring continued at room temperature for 16 hours. The reaction was concentrated in vacuo. The residue was purified on silica gel (0-10% EtOAc in hexanes) to yield **36** as a colorless oil (27.7 g, 100% yield). ^1^H NMR (400 MHz, CDCl_3_) δ ppm 8.08 (1 H, d, *J*=2.2 Hz), 7.95 (1 H, dd, *J*=8.6, 2.2 Hz), 7.35 (1 H, d, *J*=8.6 Hz), 3.95 (3 H, s), 3.16 - 3.25 (1 H, m), 2.06 - 2.14 (2 H, m), 1.76 - 1.98 (2 H, m), 1.61 - 1.70 (2 H, m), 0.98 - 1.06 (3 H, m), 0.70 (3 H, s). MS ESI (pos.) m/e: 381.1 (M+H)^+^.

**36 37**

**Methyl 2-((1*R*)-2,2-dimethylcyclopentyl)-2'-fluoro-5'-(methyloxy)-1,1'-biphenyl-4-carboxylate (37)** To a stirred solution of (*R*)-methyl 3-(2,2-dimethylcyclopentyl)-4-(trifluoromethylsulfonyloxy)benzoate (28.5 g, 75 mmol) in DMF (375 ml, 75 mmol) at 23^o^C was added 2-fluoro-5-methoxyphenylboronic acid (19 g, 112 mmol), potassium carbonate (31 g, 225 mmol), followed by tetrakis(triphenylphosphine)palladium (4 g, 4 mmol). The mixture was heated to 90 ^o^C. Stirring continued for 20 hours, after which, the reaction was cooled to room temperature, diluted with water, and extracted three times with ethyl acetate. The organic layers were combined and washed twice with brine. After drying over anhydrous sodium sulfate and filtering, the organic solvent was removed under reduced pressure. The residue was purified on silica gel (0-10% EtOAc in hexanes) to yield **37** as a colorless oil (25.00 g, 94% yield). MS ESI (pos.) m/e: 357.1 (M+H)^+^.

**37 33.2**

**(2-((1*R*)-2,2-dimethylcyclopentyl)-2'-fluoro-5'-(methyloxy)-1,1'-biphenyl-4-yl)methanol (33.2)** To a stirred solution of methyl 2-((1*R*)-2,2-dimethylcyclopentyl)-2'-fluoro-5'-(methyloxy)-1,1'-biphenyl-4-carboxylate (**37**) (29.50 g, 83 mmol) in tetrahydrofuran (414 ml, 83 mmol) at 0°C was added lithium aluminum hydride (124 ml, 124 mmol). Stirring continued for 2 hours. 1N Sodium hydroxide (aq) was then added to quench the reaction, and the mixture was then extracted with ethyl acetate. The organic layers were dried over magnesium sulfate, filtered, and concentrated in vacuo. The residue was purified on silica gel (0-20% EtOAc in hexanes) to yield **33.2** as a colorless oil (23.66 g, 87% yield). MS ESI (pos.) m/e: 346.1 (M+H_2_O)^+^.

**33.2 34**

**4-(Chloromethyl)-2-((1*R*)-2,2-dimethylcyclopentyl)-2'-fluoro-5'-(methyloxy)-1,1'-biphenyl (34)**. To a stirred solution of (2-((1*R*)-2,2-dimethylcyclopentyl)-2'-fluoro-5'-(methyloxy)-1,1'-biphenyl-4-yl)methanol (**33.2**) (23.66 g, 72 mmol) in dichloromethane (360 ml, 72 mmol) and DMF (0.56 ml, 7.2 mmol) at 0°C was added thionyl chloride (11 ml, 144 mmol). Stirring continued at room temperature for 1 hour. Concentrated in vacuo. The residue was purified on silica gel (0-10% EtOAc in hexanes) to yield **34** as a colorless oil (23.0 g, 92% yield). MS ESI (pos.) m/e: 364.1 (M+H_2_O)^+^.

**38**

**5-(2-Fluoro-3-methoxybenzylidene)-2,2-dimethyl-1,3-dioxane-4,6-dione (38).** A 500 mL round bottom flask was charged with 2-fluoro-3-methoxybenzaldehyde (available from Frontier Scientific) (28.6 g, 186 mmol), 2,2-dimethyl-1,3-dioxane-4,6-dione (available from Aldrich) (34.8 g, 241 mmol), and water (350 mL). The heterogeneous mixture was stirred for 2 h at 90 °C, cooled to room temperature, and filtered. The crude solid was triturated with ether to afford **38** (30.6 g, 58.8% yield) as a crystalline, white powder. ^1^H NMR (400 MHz, CDCl_3_) δ ppm 8.47 (1 H, s), 7.37 - 7.43 (1 H, m), 7.09 - 7.19 (2 H, m), 3.88 - 3.95 (3 H, m), 1.80 - 1.87 (6 H, m)

**38 39**

**5-(1-(2-Fluoro-3-methoxyphenyl)propyl)-2,2-dimethyl-1,3-dioxane-4,6-dione (39).** A 500 mL round bottom flask was charged with **38** (5.00 g, 17.8 mmol) and THF (100 mL) and cooled to 0 °C under N_2_. To the cold slurry was added ethylmagnesium bromide (3.0 M in ether) (available from Aldrich) (11.9 mL, 35.7 mmol) dropwise over 15 minutes. The cooling bath was removed, and the mixture was stirred for 1 h at ambient temperature. The reaction was quenched with 1 N HCl and extracted with EtOAc. The combined organics were washed with brine, dried (MgSO_4_), and concentrated to afford **39** (5.17 g, 93% yield) as a yellow oil. The crude product was used without further purification. ^1^H NMR (400 MHz, CDCl_3_) δ ppm 7.04 - 7.12 (2 H, m), 6.86 - 6.92 (1 H, m), 4.05 (1 H, dt, *J*=10.3, 4.2 Hz), 3.89 (3 H, s), 3.77 - 3.81 (1 H, m), 2.12 - 2.26 (1 H, m), 1.78 - 1.91 (1 H, m), 1.71 (3 H, s), 1.60 (3 H, s), 0.90 - 0.96 (3 H, t, *J*=7.4 Hz)

**39 40**

**3-(2-Fluoro-3-methoxyphenyl)pentanoic acid (40).** A 500 mL round bottom flask was charged with **39** (5.17 g, 16.7 mmol) and 10:1 DMF/water (80 mL). The resulting solution was stirred overnight at 90 °C, cooled to room temperature, and diluted with ether. The organics were washed with water and brine, dried (MgSO_4_), and concentrated to afford **40** (3.44 g, 91% yield) as a yellow oil. The crude product was used without further purification. ^1^H NMR (400 MHz, CDCl_3_) δ ppm 10.40 (1 H, br s), 7.01 (1 H, td, *J*=8.0, 1.6 Hz), 6.80 - 6.87 (1 H, m), 6.75 (1 H, ddd, *J*=7.8, 6.2, 1.5 Hz), 3.84 - 3.92 (3 H, m), 3.36 (1 H, dtd, *J*=9.3, 7.5, 7.5, 5.5 Hz), 2.64 - 2.77 (2 H, m), 1.70 - 1.83 (1 H, m), 1.59 - 1.70 (1 H, m), 0.82 (3 H, t, *J*=7.3 Hz)

**40 41**

**3-(2-Fluoro-3-hydroxyphenyl)pentanoic acid (41).** A 500 mL round bottom flask was charged with **40** (26.0 g, 115 mmol), N-methylpyrrolidone (250 mL), sodium hydroxide (20.7 g, 518 mmol), and 1-dodecanethiol (available from Aldrich) (96.5 mL, 403 mmol). The mixture was stirred for 5 h at 125 °C, cooled to room temperature, quenched with 1 N HCl, and diluted with EtOAc. The organics were washed with water and brine, dried (MgSO_4_), and concentrated to give a biphasic mixture. The greasy top layer was diluted with hexanes and decanted. The polar bottom layer was purified by silica gel flash chromatography (20-50% EtOAc/hexane) to afford **41** (21.5 g, 88% yield) as a colorless oil. ^1^H NMR (400 MHz, CDCl_3_) δ ppm 6.94 - 7.00 (1 H, m), 6.84 - 6.90 (1 H, m), 6.71 (1 H, td, *J*=7.1, 1.7 Hz), 5.13 (1 H, br. s.), 3.28 - 3.40 (1 H, m), 2.63 - 2.77 (2 H, m), 1.71 - 1.82 (1 H, m), 1.58 - 1.71 (1 H, m), 0.83 (3 H, t, *J*=7.3 Hz). MS ESI (pos.) m/e: 230.1 (M+H_2_O)^+^.

**41 42**

**Methyl 3-(2-fluoro-3-hydroxyphenyl)pentanoate (42).** A 500 mL round bottom flask was charged with **41** (21.5 g, 101 mmol), MeOH (300 mL), and sulfuric acid (1.08 mL, 20.3 mmol). The solution was stirred for 1 h at 80 °C, cooled to room temperature, and diluted with ether. The organics were washed with saturated aqueous NaHCO_3_, water, and brine, dried (MgSO_4_), and concentrated. The crude product was purified by silica gel flash chromatography (0-30% EtOAc/hexane) to afford **42** (20.6 g, 90% yield) as a yellow oil. ^1^H NMR (400 MHz, CDCl_3_) δ ppm 6.93 - 7.00 (1 H, m), 6.83 - 6.90 (1 H, m), 6.70 (1 H, td, *J*=7.1, 1.7 Hz), 5.14 (1 H, d, *J*=4.1 Hz), 3.56 - 3.65 (3 H, m), 3.28 - 3.40 (1 H, m), 2.58 - 2.75 (2 H, m), 1.53 - 1.80 (3 H, m), 0.82 (3 H, t, *J*=7.4 Hz). MS ESI (neg.) m/e: 225.1 (M-H)^+^.

**42 42.1 42.2**

**(*R*)-Methyl 3-(2-fluoro-3-hydroxyphenyl)pentanoate (42.1) and (*S*)-methyl 3-(2-fluoro-3-hydroxyphenyl)pentanoate (42.2).** Racemic **42** (20 g) was resolved by chiral HPLC (Chiralcel OD column, 3% IPA/hexane, detection at 220 nm) to afford (in order of elution) **42.1** (8 g, 80% yield, 99% e.e.) and **42.2** (8 g, 80% yield, 99% e.e.) as colorless oils.

**28 42.1 31**

**(*R*)-methyl 3-(3-((2-((*R*)-2,2-dimethylcyclopentyl)-2'-fluoro-5'-methoxy-[1,1'-biphenyl]-4-yl)methoxy)-2-fluorophenyl)pentanoate (31).** A mixture of (*R*)-Methyl 3-(2-fluoro-3-hydroxyphenyl)pentanoate **42.1** (0.43 g, 1.9 mmol), **28** (0.725 g, 2.09 mmol), and cesium carbonate (0.805 g, 2.47 mmol) in DMF (10 ml) was stirred overnight at room temperature. The mixture was diluted with EtOAc, washed with water and brine, dried (MgSO_4_), and concentrated. The residue was then purified by flash chromatography (SiO_2_ gel 60, eluted with 0%-20% EtOAc in hexanes). Fractions containing the desired product were combined and concentrated to provide **31** (1.02g, 100%) as a colorless oil. ^1^H NMR (500 MHz, CDCl_3_) δ ppm 7.42 - 7.49 (1 H, m), 7.30 - 7.36 (1 H, m), 7.18 - 7.25 (1 H, m), 6.96 - 7.09 (2 H, m), 6.88 - 6.93 (1 H, m), 6.82 - 6.88 (1 H, m), 6.75 - 6.81 (1.6 H, m), 6.67 (0.4 H, dd, *J*=6.0, 3.1 Hz), 5.14 - 5.20 (2 H, m), 3.80 (3 H, s), 3.62 (3 H, s), 3.49 (1 H, q, *J*=7.0 Hz), 3.34 - 3.42 (1 H, m), 2.97 (0.4 H, dd, *J*=10.3, 8.8 Hz), 2.83 (0.6 H, ddd, *J*=10.4, 8.2, 2.2 Hz), 2.63 - 2.73 (2 H, m), 2.10 - 2.20 (0.6 H, m), 1.96 - 2.07 (1.4 H, m), 1.60 - 1.84 (4 H, m), 1.48 - 1.57 (1 H, m), 1.31 - 1.41 (1 H, m), 0.83 (3 H, t, *J*=7.3 Hz), 0.66 - 0.71 (3 H, m), 0.61 (1 H, s), 0.56 (2 H, s). ESI (pos.) m/e: 559.3 (M+Na)^+^, 554.3 (M+H_2_O)^+^. ^1^H NMR shows a mixture of compounds (6:4 ratio) with different peaks in the absence of a chiral shift reagent. Without being bound to theory, it is hypothesized that restricted rotation around the biphenyl ring system due to the sterically encumbering dimethylcyclopentane substituent may lead to atropisomers.

**AM-6226**

**(*R*)-3-{3-[2-((*R*)-2,2-Dimethyl-cyclopentyl)-2'-fluoro-5'-methoxy-biphenyl-4-ylmethoxy]-2-fluoro-phenyl}-pentanoic acid (AM-6226)**. To a solution of **31** (1.1 g, 2.05 mmol) in 2:1 THF/MeOH (15 ml) was added 1 N aq. LiOH (5 ml, 5 mmol). The mixture was stirred overnight at room temperature, quenched with a slight excess of 1 N aq. HCl, and extracted with EtOAc. The combined organics were dried (MgSO_4_) and concentrated. The crude product was purified by silica gel flash chromatography (0–40% EtOAc/hexane) to afford **AM-6226** (1.02g, 95%) as a white foam. ^1^H NMR (500 MHz, CDCl_3_) δ ppm 7.42 - 7.49 (1 H, m), 7.29 - 7.36 (1 H, m), 7.18 - 7.25 (1 H, m), 6.96 - 7.09 (2 H, m), 6.87 - 6.93 (1 H, m), 6.82 - 6.87 (1 H, m), 6.75 - 6.80 (1.6 H, m), 6.67 (0.4 H, dd, *J*=5.9, 3.2 Hz), 5.14 - 5.20 (2 H, m), 3.80 (3 H, s), 3.62 (3 H, s), 3.32 - 3.43 (1 H, m), 2.97 (0.4 H, dd, *J*=10.3, 8.6 Hz), 2.83 (0.6 H, ddd, *J*=10.4, 8.2, 2.2 Hz), 2.63 - 2.74 (2 H, m), 2.09 - 2.20 (0.6 H, m), 1.96 - 2.05 (1.4 H, m), 1.60 - 1.84 (4 H, m), 1.48 - 1.54 (1 H, m), 1.32 - 1.42 (1 H, m), 0.79 - 0.87 (3 H, m), 0.65 - 0.71 (3 H, m), 0.61 (1 H, s), 0.56 (2 H, s). MS ESI (neg.) m/e: 521.2 (M-H). ^1^

**Synthesis of ^3^H AM-1638**

1. **43**

**2-(5,5-dimethyl-1-cyclopenten-1-yl)-2'-fluoro-5'-hydroxy-1,1'-biphenyl-4-carboxylic acid (43).** To a stirred solution of **26** (300.0 mg, 846 µmol) in CH_2_Cl_2_ (10 mL) at 0 °C was added boron tribromide (1.0M, 3809 µl, 3809 µmol). Stirring continued for two hours, afterwhich, pH7 buffer was added to the mixture at 0 ^o^C. The resulting mixture was extracted with dicholormethane and dried over MgSO_4_, concentrated in vacuo, and purified on silica gel (0%-20% EtOAc/hexane) to give **43**. ^1^H NMR (400 MHz, CDCl_3_) δ ppm 7.96 - 8.18 (2 H, m), 7.36 - 7.56 (1 H, m), 7.09 - 7.24 (2 H, m), 6.87 - 6.97 (1 H, m), 6.72 - 6.82 (1 H, m), 5.49 - 5.65 (1 H, m), 2.21 - 2.38 (2 H, m), 1.64 - 1.79 (2 H, m), 0.86 - 0.98 (6 H, m). MS ESI (pos.) m/e: 344.2 (M+H_2_O)^+^, 327.1 (M+H)^+^.

**43 44**

**Methyl 2-(5,5-dimethyl-1-cyclopenten-1-yl)-2'-fluoro-5'-hydroxy-1,1'-biphenyl-4-carboxylate (44).** To a flask containing **43** (75 mg, 230 µmol) in MeOH (2 mL) was added sulfuric acid (0.61 µl, 11 µmol). The resulting mixture was stirred at reflux overnight. The reaction was concentrated and then purified by silica gel chromatography (0 to 30% EtOAc/ Hexanes) to provide **44** (77.0 mg, 98%). ^1^H NMR (400 MHz, CDCl_3_) δ ppm 7.95 (1 H, dd, *J*=8.0, 1.8 Hz), 7.90 (1 H, d, *J*=1.8 Hz), 7.31 - 7.38 (1 H, m), 6.88 - 6.96 (1 H, m), 6.75 - 6.81 (1 H, m), 6.73 (1 H, dd, *J*=5.8, 3.0 Hz), 5.75 (1 H, s), 5.47 - 5.51 (1 H, m), 3.90 - 3.99 (3 H, s), 2.25 (2 H, td, *J*=7.0, 2.3 Hz), 1.67 (2 H, t, *J*=7.0 Hz), 0.87 (6 H, s). MS ESI (pos.) m/e: 358.2 (M+H_2_O)^+^, 341.2 (M+H)^+^.

**44 45**

**Methyl 2-(5,5-dimethyl-1-cyclopenten-1-yl)-2'-fluoro-5'-((tris(1-methylethyl)silyl)oxy)-1,1'-biphenyl-4-carboxylate (45)**. To **44** (77.0 mg, 226 µmol) in DMF (10 mL) was added imidazole (30.8 mg, 452 µmol) and then triisopropylsilyl chloride (58.1 µl, 271 µmol) and then allowed to stir for 4 hours. The reaction was diluted with EtOAc and washed with 1N HCl, water, and brine. The organics were dried over sodium sulfate, filtered concentrated and purified by silica gel chromatography (0 to 10% EtOAc/ Hexanes) to provide **45** (90.0 mg, 80% yield). ^1^H NMR (400 MHz, CDCl_3_) δ ppm 7.96 (1 H, dd, *J*=7.9, 1.9 Hz), 7.90 (1 H, d, *J*=1.8 Hz), 7.35 (1 H, dd, *J*=8.0, 1.0 Hz), 6.91 (1 H, t, *J*=8.9 Hz), 6.77 - 6.83 (1 H, m), 6.75 (1 H, dd, *J*=6.1, 2.9 Hz), 5.53 (1 H, s), 3.94 (3 H, s), 2.26 (2 H, td, *J*=7.0, 2.3 Hz), 1.67 (2 H, t, *J*=7.0 Hz), 1.17 - 1.31 (3 H, m), 1.07 - 1.13 (18 H, m),0.88 (6 H, s). MS ESI (pos.) m/e: 515.2 (M+H_2_O)^+^.

**45 46**

**(2-(5,5-Dimethyl-1-cyclopenten-1-yl)-2'-fluoro-5'-((tris(1,1-dimethylethyl)silyl)oxy)-1,1'-biphenyl-4-yl)methanol (46)**. To **45** (90.0 mg, 167 µmol) in THF (1 mL) at 0 °C was added lithium aluminum hydride, 1.0M solution in tetrahydrafuran (251 µl, 251 µmol). The reaction was stirred for one hour and then carefully diluted with water, extracted with EtOAc, washed with brine, dried over sodium sulfate, filtered, and concentrated to provide **46** (64.0 mg, 75.0% yield). ^1^H NMR (400 MHz, CDCl_3_) δ ppm 7.22 - 7.36 (3 H, m), 6.87 - 6.94 (1 H, m), 6.74 - 6.81 (2 H, m), 5.51 (1 H, t, *J*=2.0 Hz), 4.75 (2 H, d, *J*=5.7 Hz), 2.26 (2 H, td, *J*=7.0, 2.4 Hz), 1.77 (1 H, t, *J*=6.0 Hz), 1.67 (2 H, t, *J*=7.0 Hz), 1.19 - 1.30 (3 H, m), 1.07 - 1.14 (18 H, m), 0.90 (6 H, s)

**46 30 47**

**Methyl (3S)-3-cyclopropyl-3-(3-(((2-(5,5-dimethyl-1-cyclopenten-1-yl)-2'-fluoro-5'-hydroxy-1,1'-biphenyl-4-yl)methyl)oxy)phenyl)propanoate (47).** To flask containing **30** (30.1 mg, 137 µmol), **46** (64.0 mg, 137 µmol), and triphenylphosphine polymer supported (68.3 mg, 205 µmol) in DCM (1 mL) was added diethyl azodicarboxylate (32.3 µl, 205 µmol) at 0 °C then allowed to warm to room temperature and stirred for 1 hour. LCMS and TLC results indicated the Mitsunobu reaction was completed. Thus tetrabutylammonium fluoride (1.0M in THF, 0.164 mL, 0.164 mmol) was added. The reaction was concentrated after LCMS indicated the reaction was complete. The residue was purified by silica gel chromatography (0 to 20% EtOAc/ Hexanes) to provide **47** (66.3 mg, 94% yield). ^1^H NMR (400 MHz, CDCl_3_) δ ppm 7.37 - 7.42 (1 H, m), 7.28 - 7.33 (2 H, m), 7.24 (1 H, t, *J*=7.8 Hz), 6.83 - 6.95 (5 H, m), 6.69 - 6.75 (2 H, m), 5.51 (1 H, s), 5.09 (2 H, s), 4.69 (1 H, s), 3.62 (4 H, s), 2.68 - 2.82 (2 H, m), 2.31 - 2.41 (1 H, m), 2.25 (2 H, td, *J*=7.0, 2.3 Hz), 1.67 (2 H, t, *J*=7.0 Hz), 0.96 - 1.08 (1 H, m), 0.87 (6 H, s), 0.53 - 0.62 (1 H, m), 0.38 - 0.47 (1 H, m), 0.26 (1 H, dq, *J*=9.6, 4.8 Hz), 0.15 (1 H, dq, *J*=9.6, 4.9 Hz). MS ESI (pos.) m/e: 537.3 (M+Na)^+^, 532.3 (M+H_2_O)^+^, 515.2 (M+H)^+^.

**47 ^3^H_3_ AM-1638**

**^3^H_3_ AM-1638.** Moravek Biochemicals and Radiochemicals synthesized tritium labeled A-1638 by alkylation of **66.14E** with ^3^H_3_CI and subsequent hydrolysis.

**^3^H labeled compounds**

**Synthesis of ^3^H AMG 837**

**7 12 48**

**(R)-Ethyl 3-(4-((4'-(trifluoromethyl)-[1,1'-biphenyl]-3-yl)methoxy)phenyl)pent-4-ynoate (48). 48** was synthesized analogously to compound **8** from compounds **7** and **12**. ^1^H NMR (400 MHz, CDCl_3_) δ ppm 7.69 - 7.74 (4 H, m), 7.67 (1 H, s), 7.57 (1 H, dt, *J*=7.1, 1.8 Hz), 7.44 - 7.54 (2 H, m), 7.30 - 7.37 (2 H, m), 6.94 - 7.00 (2 H, m), 5.11 - 5.16 (2 H, s), 4.08 - 4.19 (3 H, m), 2.82 (1 H, dd, *J*=15.4, 8.3 Hz), 2.71 (1 H, dd, *J*=15.3, 7.0 Hz), 2.29 (1 H, d, *J*=2.5 Hz), 1.23 (2 H, t, *J*=7.1 Hz). MS ESI (pos.) m/e: 475.1 (M+Na)^+^, 470.0 (M+H_2_O)^+^, 453.0 (M+H)^+^.

**48 ^3^H_3_ AMG 837**

**^3^H_3_ AMG 837.** Moravek Biochemicals and Radiochemicals synthesized tritium labeled AMG 837 by alkylation of **48** with ^3^H_3_CI and subsequent hydrolysis.

**H NMR shows a mixture of compounds (6:4 ratio) with different peaks in the absence of a chiral shift reagent. Without being bound to theory, it is hypothesized that restricted rotation around the biphenyl ring system due to the sterically encumbering dimethylcyclopentane substituent may lead to atropisomers.**
